# Improved Isolation Procedures for Okadaic Acid Group Toxins from Shellfish (*Mytilus edulis*) and Microalgae (*Prorocentrum lima*)

**DOI:** 10.3390/md18120647

**Published:** 2020-12-16

**Authors:** Jane Kilcoyne, Stephen Burrell, Cíara Nulty, Rafael Salas, Elliott J. Wright, Isabelle Rajotte, Christopher O. Miles

**Affiliations:** 1 Marine Institute, Rinville, Oranmore, Co. Galway H91 R673, Ireland; stephen.burrell@marine.ie (S.B.); ciara.nulty@marine.ie (C.N.); rafael.salas@marine.ie (R.S.); 2 Biotoxin Metrology, National Research Council Canada, Halifax, NS B3H 3Z1, Canada; elliott.wright@nrc-cnrc.gc.ca (E.J.W.); isabelle.rajotte@nrc.ca (I.R.); christopher.miles@nrc-cnrc.gc.ca (C.O.M.)

**Keywords:** OA group toxins, DSP, purification, shellfish, *Prorocentrum lima*, marine biotoxins, LC-MS, NMR

## Abstract

Okadaic acid (OA) group toxins may accumulate in shellfish and can result in diarrhetic shellfish poisoning when consumed by humans, and are therefore regulated. Purified toxins are required for the production of certified reference materials used to accurately quantitate toxin levels in shellfish and water samples, and for other research purposes. An improved procedure was developed for the isolation of dinophysistoxin 2 (DTX2) from shellfish (*M. edulis*), reducing the number of purification steps from eight to five, thereby increasing recoveries to ~68%, compared to ~40% in a previously reported method, and a purity of >95%. Cell densities and toxin production were monitored in cultures of *Prorocentrum lima*, that produced OA, DTX1, and their esters, over ~1.5 years with maximum cell densities of ~70,000 cells mL^−1^ observed. Toxin accumulation progressively increased over the study period, to ~0.7 and 2.1 mg L^−1^ of OA and DTX1 (including their esters), respectively, providing information on appropriate harvesting times. A procedure for the purification of OA and DTX1 from the harvested biomass was developed employing four purification steps, with recoveries of ~76% and purities of >95% being achieved. Purities were confirmed by LC-HRMS, LC-UV, and NMR spectroscopy. Additional stability observations led to a better understanding of the chemistry of these toxins.

## 1. Introduction

**Figure 1:**
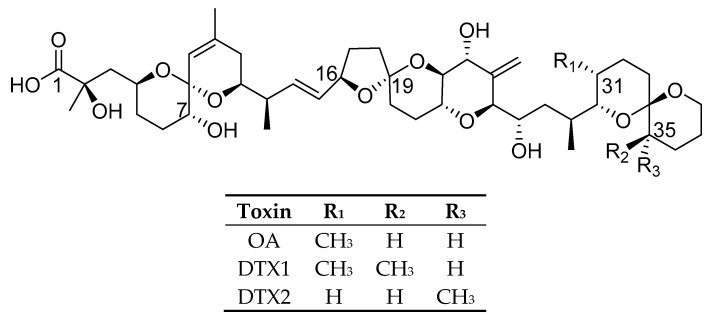
Structures of major okadaic acid (OA) group toxins.

Okadaic acid (OA) group toxins (Figure 1) are lipophilic polyether toxins produced by the genera *Prorocentrum* [1,2] and *Dinophysis* [3,4] that can lead to diarrhetic shellfish poisoning (DSP) events. The first poisoning incident associated with this toxin group occurred in Japan in 1976, following the consumption of mussels [5]. The OA group is comprised principally of okadaic acid (OA), dinophysistoxin 1 (DTX1), dinophysistoxin 2 (DTX2), and their esters [4]. The esters can be produced by the microalgae (*Dinophysis* and *Prorocentrum* spp.) [6,7,8,9], or by the shellfish via esterification of the toxins [10].

The EU regulatory limit is set at 160 µg kg^−1^ for OA, DTX1, DTX2, and their esters [11], with the EU regulatory method of analysis being LC-MS/MS [12]. The current toxic equivalent factors (TEFs) applied in the regulation (OA = 1, DTX1 = 1, DTX2 = 0.6) are based on intraperitoneal studies; however, a recent mouse oral study assessing toxicity suggested TEFs of OA = 1, DTX1 = 1.5, DTX2 = 0.3 [13]. Another study using the in vitro neuro-2a bioassay found that DTX1 is more potent than OA, while DTX2 is less potent [14].

Detection of OA group toxins in shellfish samples has resulted in significant shellfish farm closures worldwide [4,15]. In Ireland, OA group toxins are detected in shellfish annually, predominantly arising from blooms of *Dinophysis acuta* and *Dinophysis acuminata*, leading to regular shellfish harvesting restrictions, particularly during the summer months [16].

OA group toxins are serine/threonine protein phosphatase inhibitors; however, the observed harmful effects of these toxins (not relating to DSP) cannot be explained by protein phosphatase inhibition alone, and therefore more research into the mechanism of action is warranted [17,18]. As serine/threonine protein phosphatases play an important role in cellular processes, OA is used as a tool in the study of various human diseases that are linked to their dysregulation (e.g., Alzheimer’s disease) [19,20]. 

The availability of highly pure toxin for the production of certified reference materials (CRMs) is essential to ensure that monitoring programs can accurately quantitate levels of these toxins in shellfish and water samples, and for ensuring reliable quantitative toxicological evaluations. Pure compounds are further required for developing new analytical methods, and for use in other areas of research (e.g., pharmacology, the development of biosensors, etc.). Efficient isolation procedures are therefore necessary to provide these compounds cost-effectively and in sufficient purity and quantity to permit toxicological studies and method development.

Many *Prorocentrum* spp. produce OA, DTX1, and their diol esters [21], while *Dinophysis* spp. can produce OA, DTX1, DTX2, their diol esters, and pectenotoxins (PTXs) [4]. The toxin profiles can vary between location and strain. A recent study reported the full metabolomic profile of Galacian strains of *D. acuta* and *D. acuminata* by LC-MS/MS. The complex and diverse chemical profiles differed significantly between species, feeding regimes, and prey organisms [22].

*Prorocentrum* spp. are relatively straightforward to culture and can produce relatively high amounts of toxin per cell (pg cell^−1^). The *Dinophysis* spp., however, are much more difficult to grow because they require a ciliate to feed on, which in turn needs to be fed with a cryptophyte [23]. OA, DTX1, and PTX2 were isolated via large-scale cultivation of *D. acuminata* [24]. However, to date, there are no reports of DTX2 purification from *Dinophysis* culture biomass. Sources of DTX2 for purification have come from shellfish (*Mytilus edulis*) [25,26,27], and in situ harvesting of a *D. acuta* bloom [28]. OA and DTX2 have been synthesised, but synthesis requires multiple steps, with low yields (<3%) [29,30].

The methods reported to date describe up to eight- and six-step procedures for the purification of OA/DTXs from shellfish and microalgae, respectively [26,27,31]. In this paper, we describe efforts made, as part of the MARBioFEED project [32], to enhance the purification methods for the isolation of these toxins and to further assess toxin stability.

## 2. Results and Discussion

### 2.1. Isolation of DTX2 from Shellfish

**Figure 2:**
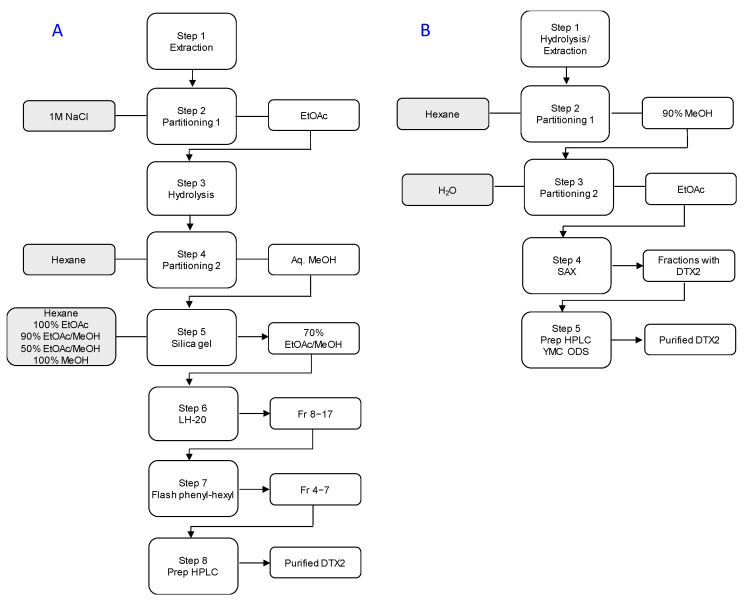
Two methods for isolation of dinophysistoxin-2 (DTX2) from shellfish: (**A**) the procedure of Beach et al. [26], and; (**B**) the newly-developed improved method.

A method published in 2016 describes an eight-step procedure for purification of DTX2 from shellfish, with recoveries of ~40% [26]. Once extracted, the sample underwent a partitioning between water and EtOAc, followed by hydrolysis (to convert the esterified forms back into DTX2), and a second partitioning between hexane and aqueous MeOH. The sample was further purified using column chromatography on silica gel, LH-20, and phenyl-hexyl phases before using semi-preparative reverse-phase HPLC with a neutral mobile phase (Figure 2A).

Efforts were made to improve procedural efficiencies and yields. The shellfish used in this study were the same as those used in the Beach et al. study [26]. The hepatopancreas (in which the toxins are concentrated) was dissected, homogenised, and freeze dried. LC-HRMS analysis of a hydrolysed extract showed that a significant proportion (~75%) of the total DTX2 present in the sample was in the esterified form. The initial extraction step was combined with base-hydrolysis to convert the DTX2 esters back into the parent compound. This led to a more efficient extraction and eliminated one step from the previously reported procedure [26]. The first two partitioning steps in the method reported by Beach et al. [26] were performed in reverse order, which also reduced the number of evaporation steps required. Following the partitioning between hexane and aqueous MeOH, the sample was evaporated and taken up in MeOH, resulting in the precipitation of salts, which were removed by filtration. The sample was then evaporated and partitioned between water and EtOAc. This had the effect of enhancing the clean-up (via reduction in the sample mass) by 81% (five-fold) compared to the previously reported procedure [26]. A similar enhanced clean-up was also obtained in separate azaspiracid (AZA) isolations where the first two partitioning steps [33] were reversed (data not shown).

Method development trials were then performed comparing alumina (using the method described in Section 3.4) and strong anion exchange (SAX) chromatography. A significantly improved clean-up was achieved using SAX chromatography (Figure S1), and a method was developed such that a clean-up of ~94% was achieved with a recovery of >95%. Prior to loading the sample onto the SAX stationary phase, the sample was taken up in 3:7 MeOH-H_2_O, to which ammonium bicarbonate was added. The ammonium bicarbonate enabled complete dissolution of the sample. The toxin was eluted using a pH-gradient (from pH 3.0 to 2.6, in steps of 0.2 pH units) of acidic water. The sample was then sufficiently pure for semi-preparative HPLC (Figure S1B).

The enhanced DTX2 clean up achieved in this study, by reversing the first two partitioning steps and adding SAX chromatography, enabled the silica gel, LH-20, and flash phenyl-hexyl chromatographic steps reported in the Beach et al. [26] procedure to be eliminated (Figure 2B and Table 1). The Beach et al. [26] method employed a neutral mobile phase for the final purification step (semi-preparative HPLC). Using such a mobile phase gives rise to poorly shaped peaks and separation from other sample contaminants. The method was amended in the present study to use an acidic (pH 3.3) mobile phase, which resulted in good peak shape and sufficient separation of the DTX2 from other sample impurities.

The organic solvent was evaporated from the combined DTX2 fractions and the pH of the sample was adjusted to 7 using NaOH, to reduce the effects of acid instability (see below). The DTX2 was recovered on solid-phase extraction (SPE) cartridges to remove any buffers and salts in the sample but also to reduce the water content in, and volume of, the DTX2 fractions prior to evaporation. This SPE recovery resulted in very little loss of toxin, with recoveries of >98% being achieved.

**Figure 3:**
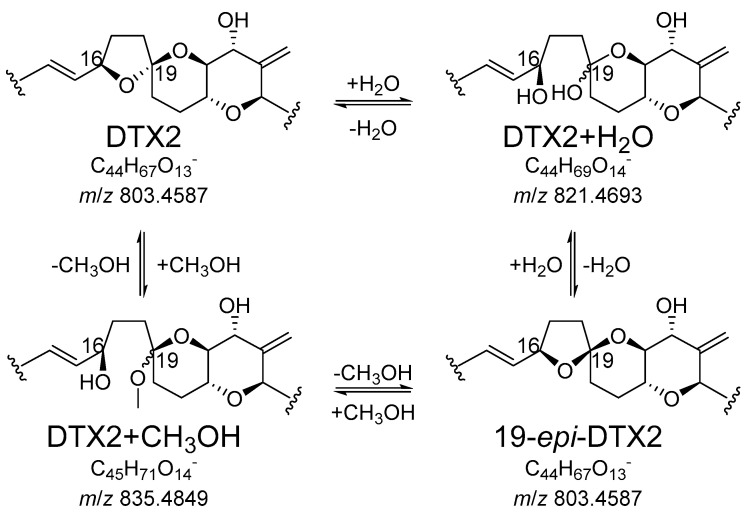
Scheme showing a possible route for interconversion of DTX2 and 19-*epi*-DTX2 via ring-opening of the ketal at C-19 (a standard ketal-hemiketal equilibration reaction), mediated by water and MeOH. The *m*/*z* values and elemental compositions are for the corresponding [M − H]^−^ ions.

The purification of DTX2 is complicated by the fact that it can form an isomeric degradation product (19-*epi*-DTX2). The 19-epimer elutes at the same time as OA (using the methods described in this study), and hence it is desirable that the levels of this epimer remain as low as possible, so as not to interfere with the analysis of OA when a mixed CRM solution is prepared. Purity analysis of DTX2 by LC-HRMS and LC-UV indicated purities were ~95% (Table 1 and Table 2; Figures S2–S4). The 19-epimer of DTX2 was present at ~2.4% (Table 2; Figures S2 and S3), so a preliminary investigation of the kinetics of the 19-epimerisation reaction was conducted using LC-MS/MS with a neutral mobile phase to minimise potential on-column isomerisation reactions. This showed that epimerisation was not measurable in neutral MeOH at 10 °C overnight, whereas epimerisation followed pseudo first-order kinetics with a half-life of 2.3 h in MeOH containing 1% *v*/*v* formic acid at 10 °C (Figure S5). Extrapolation of the kinetic data indicated that at equilibrium, 2–3% of the DTX2 would be present in the form of its 19-epimer under these conditions, and given their structural similarities, OA and DTX1 are likely to be similarly susceptible to acid-catalysed epimerisation. In addition, two earlier-eluting peaks, with *m*/z consistent with DTX2 + H_2_O, were also detected that together comprised ~0.9% (Table 2 and Figures S2 and S3) by LC-HRMS. Similarly, DTX2 + MeOH isomers and very low levels of DTX2 + CD_3_OH isomers (which must have been produced during NMR analysis) were subsequently detected by LC-HRMS. These observations are consistent with the mechanisms shown in Figure 3, the rate and extent of which will vary with solvent, pH, temperature and length of storage. Other ketal centres in DTX2 probably also undergo epimerisation, given the detection of at least four MeOH derivatives after storage and shipment of the sample, but these other reactions are probably slower, given that only two peaks containing CD_3_OH-derivatives were observed after ^1^H NMR analysis (Figures S6–S10). In a previous study, isomerization of AZA1 was observed during NMR analysis in CD_3_OD but appeared to cease upon addition of 0.1% *v*/*v* d5 pyridine [34], suggesting that such isomerisations are also acid-catalysed.

A contaminant was visible in the LC-UV (210 nm) trace (Figure S4) that eluted just after DTX2, but this was not detected by LC-HRMS and was not identified. ^1^H NMR spectroscopy did not reveal the presence of any major impurities, although minor olefinic resonances were detectable with a total relative intensity of ~5% for DTX2 (Figures S6–S10), closely paralleling purity estimates based on LC-HRMS. Unfortunately, 1D ^1^H NMR spectroscopy is not ideal for detecting small amounts of contaminants that are closely related structurally to the main component, as the majority of the resonances of the contaminants are very similar to those of the major component. Overall, the method was reduced from an eight- to a five-step procedure, with recoveries increased from ~40 to ~68%, relative to the method of Beach et al. [26], and with a purity of >95% (Figure 2, Table 1).

### 2.2. Isolation of OA and DTX1 from Prorocentrum lima

#### 2.2.1. *P. lima* Culturing

**Figure 4:**
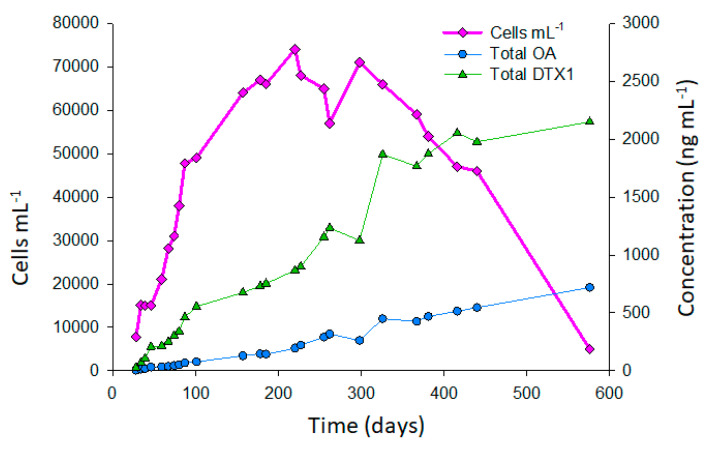
*P. lima* cell counts and toxin accumulation over ~1.5 years.

A study was performed on the growth and toxin accumulation of *P. lima* strain CCMI-1036 over ~1.5 years, and the results show maximum cell densities (~70,000 cells mL^−1^) were achieved after ~5 months of growth (Figure 4), after which cell counts plateaued for ~7 months, and then started to decline. Over the study period, toxin concentrations continued to increase steadily up until ~11 months (381 d) of growth. After 381 d and up until 576 d (the final 6.5 months of the study) the increase in toxin levels was less significant.

The culture produced OA, DTX1, and their esters, with the esters of both OA and DTX1 comprising ~40‒50% of the total toxin content. *P. lima* strains typically produce OA as the predominant toxin [15], but for this strain, DTX1 was dominant, being ~3-fold higher than OA (Figure 4). Although the growth was slow (0.028 d^−1^), toxin accumulation was relatively high, with ~0.7 and 2.1 mg L^−1^ OA and DTX1 (including their esters), respectively, detected after ~1.5 years. 

A previous study of the growth and toxin accumulation in *A. spinosum* showed that AZAs were adsorbed onto the culture flask surface, once the cells lysed and the toxin was released into the medium, leading to a decline in toxin concentrations over the sampling period [35]. The same culture flasks were used in this study, and no decline in toxin concentrations was observed, indicating that adsorption was not an issue for the OA group toxins. In a previous study, however, it was noted that OA derivatives (especially 7-deoxyOA) are very liable to be adsorbed to the walls of plastic containers when performing protein phosphatase inhibition studies [36]. 

#### 2.2.2. Purification

A previously published method [26] described a six-step procedure for purification of OA from *Prorocentrum concavum* (which mainly produces OA) biomass. Although no semi-preparative HPLC was required in this procedure, notable losses of toxin occurred after the initial large-scale vacuum-assisted silica LC step. In the same study, DTX1 was purified from *P. lima* biomass, also in a six-step procedure, using semi-preparative HPLC as the final clean-up step [26].

In the new method, the culture was treated with base to hydrolyse ester derivatives to the parent compounds, eliminating the need to perform a separate cell lysis step. Following neutralization, HP-20 resin was added to adsorb the toxins, and the recovered resin was eluted. Initial trials to assess the use of a SAX stationary phase, as in the method employed for the isolation of DTX2 from shellfish, resulted in significant losses of toxin (~40%) (data not shown). As an alternative, an alumina stationary phase was assessed and found to produce superior recoveries (~90%), and acted as an efficient clean-up (~85%) step (Table 3). This stationary phase was found to be effective previously in the purification of OA and DTX2 from HP-20 resin extracts [28]. The sample was then chromatographed on LH-20, resulting in a clean-up of ~60%, and recoveries of ~95%. As in the purification of DTX2, the final semi-preparative HPLC step employed an acidic (pH 3.3) mobile phase that resulted in good peak shape and sufficient separation of OA and DTX1 from other sample impurities. Recoveries were good (~90%), indicating that there were no major acid instability issues (Table 3).

Purity analysis of the OA by LC-HRMS and LC-UV indicated that purities were >95% (Table 4; Figures S11–S13). The 19-epimer of OA (~1.4%) was present, as was another isomer (<1%) that eluted prior to the 19-epimer (Table 4; Figures S11 and S12). DTX1 isomers eluted just after the OA peak in the semi-preparative HPLC, that likely contaminated the OA fractions, and this explains the presence (<1%) of DTX1 (due to subsequent equilibration of the DTX1 isomers back to DTX1) in the final purified sample of OA (Table 4; Figures S11 and S12). In light of the subsequent stability study on DTX2 (Section 2.1), it is possible that these DTX1 isomers arose through acid-catalysed isomerisation of DTX1 during purification. In addition, two isomeric peaks of OA + MeOH were also detected in the sample (Table 4; Figures S11 and S12). ^1^H NMR analysis of the OA was consistent with the high purity of this material. Minor olefinic signals were present in the same region as for the DTX2 (Figures S6–S10), but these amounted to only ~1% of the intensity of the main olefinic signals, consistent with the results of the LC-HRMS analyses.

Purity analysis of the DTX1 by LC-HRMS and LC-UV indicated that purities were >95% (Table 5; Figures S14–S16). The 19-epimer of DTX1 (~2%) was present, as was another isomer (~0.6%) that eluted prior to the 19-epimer (Table 5; Figures S14 and S15). An additional peak was present (0.09%) in the LC-HRMS in the purified DTX1 sample that corresponded to 7-deoxyOA (Table 5 and Figures S14 and S15), a compound that has previously been reported in *P. lima* [36,37]. LC-HRMS analysis also showed the presence of trace levels of DTX1 + MeOH (0.08%). As with OA, ^1^H NMR analysis was consistent with DTX1 at high purity (Figures S6–S10), and indicated only low levels of related contaminants.

**Figure 5:**
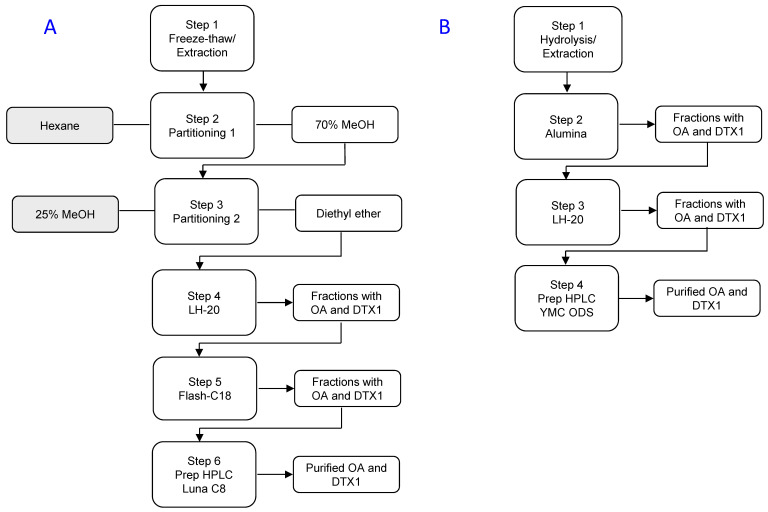
Isolation of OA and dinophysistoxin 1 (DTX1) from *P. lima* showing: (**A**) the procedure published by Beach et al. [26], and; (**B**) the improved method.

Overall, a method was developed that employed a four-step procedure (Figure 5), with recoveries of ~76% and purities of 98.2 and 97.2% (assuming 1:1 relative molar responses for the detected impurities) for OA and DTX1, respectively (Table 4 and Table 5).

Given the susceptibility to acid-catalysed isomerisation of these toxins, it would be advisable to avoid exposure to acids in the late stages of purification of OA/DTXs. The formation of isomers would be reduced under neutral conditions in the final semi-preparative HPLC step; however, a neutral mobile phase results in broader peaks and poorer resolution from impurity peaks, thereby leading to reduced efficiencies and yields.

## 3. Materials and Methods 

### 3.1. Reagents

Solvents (LC-MS grade) were from Labscan (Dublin, Ireland) and Fisher Scientific (Whitby, ON, Canada). Distilled water was further purified using the Barnstead nanopure diamond UV (Thermo Scientific, Des Moines, IA, USA) and Milli-Q (Millipore Corp., Billerica, MA, USA) purification systems. Formic acid (≥98%), ammonium formate (>98%), sodium hydroxide, Diaion HP-20 polymeric resin (≥0.25 mm), ammonium acetate (97%), and SAX were from Sigma-Aldrich (Steinheim, Germany and Oakville, ON, Canada). Formic acid (98%) was from Honeywell (Oakville, ON, Canada). Hydrochloric acid was from VWR (Fontenay-sous-bois, France), while ammonium bicarbonate (99%) and ammonium hydroxide were from Acros (Trenton, NJ, USA). Aluminium oxide 90 active basic and acetic acid were from Merck (Darmstadt, Germany). Sephadex LH-20 was from GE Healthcare (Uppsala, Sweden). CRMs OA-d, DTX1-b, and DTX2-b were from the National Research Council (Halifax, NS, Canada) [26].

### 3.2. Isolation of DTX2 from Shellfish

Cooked whole-mussel tissue from *M. edulis* collected in 2010, from the southwest of Ireland, was dissected to yield ~400 g of hepatopancreas, which was homogenised and freeze-dried (117 g). MeOH (500 mL) and 2.5 M NaOH (62.5 mL) in 90% MeOH was added to the sample, and sonicated for 5 min. The sample was then placed in a water bath set at 76 °C for 30 min. Once the sample cooled, 2.5 M HCl (62.5 mL) in MeOH was added. The suspension was filtered, and the retentate further extracted with MeOH (3 × 500 mL) using a Waring blender. The four methanolic extracts were combined and evaporated in vacuo. The residue was partitioned between hexane (200 mL) and MeOH-H_2_O (9:1, 200 mL). The MeOH-H_2_O layer was evaporated in vacuo and the residue dissolved in EtOAc (200 mL) and partitioned with H_2_O (200 mL). The EtOAc layer was evaporated in vacuo and the residue dissolved in 10:3 H_2_O-MeOH (28.5 mL), containing 0.7 g ammonium bicarbonate, and loaded onto a strong anion exchange (SAX) column (20 g). The column was washed with H_2_O (~50 mL) until the eluate was colourless. The DTX2 was then eluted using a stepped gradient of acidic (formic acid) H_2_O at pH 3 (1 × 50 mL), pH 2.8 (1 × 50 mL) and pH 2.6 (8 × 50 mL), analysing each fraction by LC-HRMS (Section 3.5.1). The fractions (4–10, pH 2.6) containing the toxin were combined (~350 mL), and the pH adjusted to 7 using NaOH. The DTX2 was recovered by partitioning with CH_2_Cl_2_. Final purification of DTX2 was achieved by semi-preparative HPLC (Shimadzu 10AVp; Kyoto, Japan), with photodiode array (PDA) detection (210 nm), using a YMC-Pack ODS-AQ (250 × 10 mm, 5 µm; YMC, Kyoto, Japan) column eluted with 0.9:1 CH_3_CN-H_2_O (pH adjusted to 3.3 using formic acid) at 4 mL min^−1^ for 65 min. The column was flushed with MeOH for 5 min, returned to the initial conditions and held for 5 min to equilibrate the system. The column temperature was 30 °C. The organic solvent was evaporated in vacuo from the combined DTX2 fractions, and the pH of the aqueous sample was adjusted to 7 using NaOH, to limit acid instability.

Purified DTX2 was recovered by loading the aqueous sample dropwise onto a solid-phase extraction cartridge (Oasis HLB, 200 mg), washing with H_2_O (20 mL) to remove the buffer, and eluting with MeOH (25 mL). Removal of solvent by evaporation *in vacuo* afforded purified DTX2 (7.3 mg) as a white solid.

#### 19-*epi*-DTX2 Kinetics

An aliquot of 19-*epi*-DTX2 available from previous work [26] was dissolved in MeOH (50 µL) in an LC vial insert, formic acid (0.5 µL) was added, and the vial was placed in the sample tray and analysed periodically by LC-MS/MS. A sample of 19-*epi*-DTX2 in MeOH without acidification was used as a reference standard. Kinetic data were analysed with SigmaPlot 12.5 (Systat, San Jose, CA, USA) by fitting to a 3-parameter exponential decay curve (Figure S5).

LC-MS/MS analysis was performed on an Agilent 1260 LC (Palo Alta, CA, USA) coupled to an AB-Sciex (Concord, ON, Canada) 4000 QTRAP MS equipped with a turbospray ionization source. The autosampler temperature was maintained at 10 °C and 2 µL injection volumes were used. A Luna C18(2)HST column (50 × 2 mm, i.d., 2.5 µm; Phenomenex, Torrance, CA, USA) maintained at 20 °C was eluted at 300 µL min^−1^ with a 7 min linear gradient of 15–100% B, using 5 mM ammonium acetate in H_2_O (A) and 95% CH_3_CN (B) at pH 6.8 as the mobile phase. MS detection used selected reaction monitoring with negative polarity electrospray ionization as follows; temperature 500 °C; curtain gas 30 psi; −4.5 kV spray voltage; GS1 40 psi; GS2 60 psi; DP −50 V; and CE values of −65 and −95 eV for the 803.5→255.1 and 803.5→113.1 transitions, respectively, each analysed with a 65 ms dwell time.

### 3.3. *P. lima* Culturing

Cultures of a *P. lima* strain (CCMI-1036), isolated from the southwest coast of Ireland, were grown in 5 L (Corning cellSTACKs, Lowel, MA, USA) culture flasks, each containing ~2.2 L of L1 culture medium [38] (at 18 °C, photoperiod 12:12 light:dark), for ~1.5 years (with no addition of nutrients over the study period).

#### Culture Sampling 

For cell densities, the cultures were sampled by transferring 500 µL of well-mixed culture into a 1.5 mL centrifuge tube containing 400 µL seawater and 100 µL Lugol’s iodine. As cultures became denser, the ratio of seawater to culture was increased. Cells were counted on a Sedgwick rafter by visual microscopy (Olympus model BX53, Madison, WI, USA). Growth rate, as the exponent of the exponential equation, was calculated from cell density time series data by exponential regression of cell counts versus time for a defined period of exponential growth (day 46‒87) using Microsoft Excel (2016).

For LC-HRMS analysis of OA and DTX1, the cultures were sampled in duplicate, one for analysis of the free toxins and the other for total toxins, i.e., including esters. Into two HPLC vials, containing 900 µL MeOH, 100 µL of well-mixed culture was added, vortex-mixed for 0.5 min, and stored at −18 °C until analysis. Prior to analysis, the samples taken for total toxin were hydrolysed to convert the esters to the parent compounds by addition of NaOH (2.5 M; 125 µL), then heated in a water bath (76 °C; 20 min), cooled, and neutralised with HCl (2.5 M; 125 µL).

### 3.4. Isolation OA and DTX1 from *P. lima*

The contents of two cell stacks (~4.5 L) were transferred to a glass Erlenmeyer flask. To convert OA and DTX1 esters, 500 mL of 2.5 M NaOH was added. After 24 h, once full conversion of the esters had occurred, as indicated by LC-HRMS analysis, the sample was neutralised using 2.5 M HCl. The sample was filtered through a 20 µm mesh. LC-HRMS analysis of both the filtrate and biomass showed that >95% of the toxins were present in the filtrate. Activated HP-20 resin (50 g loosely packed into a 20 µm mesh bag) was placed in the filtrate and left to stir for 2 d, after which time >95% of toxin had adsorbed onto the resin. The resin was rinsed with water and dried. Toxins were eluted by sonicating for 0.5 h in 50 mL MeOH (×5), filtering through Whatman filter paper, and the solvent evaporated in vacuo to give an oily residue (0.32 g).

The residue was dissolved in a minimum amount of MeOH and loaded onto 15 g of alumina slurried in CH_2_Cl_2_. The column was eluted with 1:1 MeOH-CH_2_Cl_2_, MeOH, 4:1 MeOH-H_2_O, 1:1 MeOH-H_2_O, and 1:1 MeOH-H_2_O (pH 11 ammonium hydroxide) (50 mL of each). The aqueous fractions (4:1 MeOH-H_2_O and 1:1 MeOH-H_2_O, eluates 3–9) containing the toxins were combined (350 mL), and the MeOH removed by evaporation in vacuo. The pH of the remaining aqueous layer was adjusted from 10.2 to 7 with 0.1 M acetic acid. The OA and DTX1 were recovered by loading onto a solid-phase extraction cartridge (Oasis HLB, 200 mg), washing with H_2_O (20 mL) to remove the buffer, and eluting with MeOH (25 mL). The dry residue (~0.02 g) was loaded onto an LH-20 column (22 × 2.7 cm), and eluted with MeOH, with the first 15 min discarded as waste, and 3-min fractions collected thereafter. Toxins eluted in fractions 4‒6. These fractions were combined (~9 mL), and the MeOH removed by evaporation in vacuo. Final purification of OA and DTX1 was achieved by semi-preparative HPLC (Shimadzu 10AVp), with UV detection (210 nm), using a YMC-Pack ODS-AQ (250 × 10 mm, 5 µm) column, eluted with 1:1 CH_3_CN-H_2_O (pH 3.3, formic acid) at 4 mL min^−1^. The column temperature was 30 °C. The organic solvent was evaporated from the combined fractions, and the pH of the remaining aqueous residue was adjusted to 7 using NaOH to limit acid instability. The toxins were recovered by loading the aqueous samples dropwise onto SPE cartridges (Oasis HLB, 200 mg), washing with H_2_O (20 mL) to remove the buffer, and eluting with MeOH (25 mL). Removal of solvent by evaporation in vacuo afforded purified OA (2.5 mg) and DTX1 (8 mg) as white solids.

### 3.5. LC-HRMS

#### 3.5.1. Acidic Method

Analysis was performed using a Waters Acquity UPLC coupled to a Xevo G2-S QToF monitoring in MS^e^ mode (negative ionization, 100–1200 *m*/*z*), using leucine enkephalin as the reference compound. The cone voltage was 40 V, collision energy was 50 eV, the cone and desolvation gas flows were set at 0 and 600 L h^−1^, respectively, and the source temperature was 120 °C.

Chromatography was performed with an Acquity UPLC BEH C18 (50 × 2.1 mm, 1.7 µm) column (Waters, Wexford, Ireland). Binary gradient elution was used, with mobile phase A consisting of H_2_O and mobile phase B of CH_3_CN (95%) in H_2_O (both containing 2 mM ammonium formate and 50 mM formic acid). The gradient was from 5 to 90% B over 2 min at 0.3 mL min^−1^, held for 1 min, and returned to the initial conditions and held for 1 min to equilibrate the system (total run time 4 min). The injection volume was 2 µL and the column and sample temperatures were 25 and 6 °C, respectively. Quantitation using CRMs was performed using Targetlynx software. Purities were calculated based on the assumption of 1:1 relative molar responses for the detected impurities.

#### 3.5.2. Neutral Method

Analysis was performed with a Q Exactive-HF Orbitrap mass spectrometer equipped with a HESI-II heated electrospray ionization interface (ThermoFisher Scientific, Waltham, MA, USA) using an Agilent 1200 LC system including a binary pump, autosampler (sample tray 10 °C) and column oven (Agilent, Palo Alto, CA, USA). Analyses were performed with a Synergi Max-RP C12 column (50 × 2 mm, 2.5 µm; Phenomenex, Torrance, CA, USA) held at 25 °C with mobile phases A and B of H_2_O and 95:5 CH_3_CN-H_2_O, respectively, each of which contained NH_4_OAc (5 mM). Linear gradient elution (0.3 mL min^−1^) was from 15 to 100% B over 7 min, held at 100% B (4 min), then returned to 15% B over 0.1 min, with a hold at 15% B (3.9 min) to equilibrate the column (total run time 15 min). The injection volume was 5 µL. The mass spectrometer was operated in negative mode and calibrated from *m/z* 69 to 1780 with the spray voltage −2.7 kV, probe heater temperature 300 °C, capillary temperature 350 °C, and with sheath and auxiliary gas flow rates of 40 and 15 units, respectively, and MS data acquired from 2 to 11 min. Mass spectral data were collected in alternating full-scan/data-independent acquisition (FS/DIA) scan mode using an FS scan range of *m*/*z* 200–1200, a resolution setting of 60,000, AGC target of 1 × 10^6^ and max IT of 200 ms. The DIA data were collected using a resolution setting of 15,000, AGC target of 1 × 10^5^, max IT set to 100 ms and normalised collision energy 35 eV. Isolation windows were 25 *m*/*z* wide and centred at *m/z* 612, 635, 658, 682, 705, 728, 752, 775, 798, 822, 845, 868, 892, 892, and 938. DIA chromatograms were extracted (±5 ppm) for product ions at *m*/*z* 255.1238 and 239.1289.

### 3.6. LC-UV 

Purified samples (~0.5 mg mL^−1^, 10 µL) were injected onto a LC (Shimadzu 10AVp), with UV detection (210 nm), using a Luna phenyl-hexyl (250 × 4.6 mm, 5 µm; Phenomenex, Torrance, CA, USA) column eluted with 0.9:1 CH_3_CN-H_2_O (pH 3.3, formic acid) at 1 mL min^−1^. The column temperature was 30 °C.

### 3.7. NMR Spectroscopy

Samples were received in MeOH and transferred to pre-weighed scintillation vials, rinsing the ampoules with MeOH (3 × 0.5 mL). The solvent was evaporated under a stream of dry N_2_, weighed, dissolved in CD_3_OD (750 µL, 99.5 atom % D; Cambridge Isotope Laboratories, Andover, MA, USA), and transferred to NMR tubes (5 mm i.d.). ^1^H NMR spectra were acquired on a Bruker DRX-500 NMR spectrometer at 500.13 MHz (64 scans, 20 °C) using Icon NMR in Bruker TopSpin 3.2, and data were processed with TopSpin 3.6.2.

## 4. Conclusions

Methods for the purification of OA group toxins from shellfish (*M. edulis*) and microalgae (*P. lima*) were enhanced, leading to a reduction in the number of purification steps and an increase in yields. From 400 g of contaminated *M. edulis* hepatopancreas, 7.3 mg of DTX2 was purified using a five-step procedure, involving partitioning and column chromatography, with yields of ~68%. A comparison of alumina and SAX stationary phases showed that improved purification was achieved using a SAX stationary phase, allowing progression to the final semi-preparative HPLC clean-up.

The growth and toxin production of an Irish strain of *P. lima* was monitored over 1.5 years, showing an increase in OA, DTX1, and their esters (that comprised ~50% of the total toxin content) over the testing period. The results indicate that optimum harvesting time occurs after ~1 year of growth. From 4.5 L of culture (harvested after 1.5 years), 2.5 mg of OA and 8 mg of DTX1 were purified, using a four-step purification procedure, with yields of ~76%. A comparison of alumina and SAX stationary phases showed higher yields were achieved using an alumina stationary phase, with sufficient purification to allow progression to semi-preparative HPLC clean-up. 

An acidic method was used in the final semi-preparative HPLC step for all toxins, leading to greater efficiencies and higher yields. However, as these toxins are susceptible to acid-catalysed isomerization, impurities, in the form of isomers, were detected in the purified samples. Although replacing an acidic mobile phase with a neutral mobile phase at this stage may decrease the formation of such isomers, efficiencies and yields would be reduced.

The focus of this study was on the OA group toxins; however, future work could focus on describing the full metabolome and identification of other novel compounds produced by *P. lima*, that may have useful bioactive properties. The compounds purified in this study can be used to sustain supplies for the development of CRMs, and other areas of research such as toxicology and pharmacology.

## Figures and Tables

**Table 1:** Batch summary for isolation of DTX2 from shellfish, as assessed by LC-HRMS (Section 3.5.1).

	Weight (mg)	Clean-up (100 × (W_i_ − W_f_)/W_i_) (%)	DTX2 (mg)	Step Recovery (%)	Purity (%)
**Wet weight (g)**	422,000		^ 10.7		
**Dry weight (g)**	118,000		^ 10.7		
Step 1 (hydrolysis/extraction)	53,000		10.5		0.02
Step 2 (hexane partitioning)	28,000	47.2	10.3	98.1	0.04
Step 3 (EtOAc partitioning)	3300	88.2	9.9	96.1	0.30
Step 4 (SAX chromatography)	200	95.6	8.4	84.8	4.20
Step 5 (semi-prep HPLC)			7.3	86.9	* 96.7
% Recovery			68.2		

W_i_ = initial weight, W_f_ = final weight, ^ Total DTX2 (including esters) * See Figure S2.

**Table 2:** LC-HRMS (Section 3.5.2) analysis of purified DTX2 (after NMR analysis, Figure S3).

Toxin	OA + H_2_O		OA + MeOH	DTX2 + CD_3_OH	19-*epi*-DTX2	DTX2
Accurate mass (*m*/*z*) *	821.4700	821.4716	835.4857	838.5030	803.4590	803.4587
**Δ *m/z* (ppm)**	0.9	2.8	0.9	−0.9	0.4	0.0
**Retention time (min)**	3.58	3.95	4.1–4.65	4.2–4.4	4.64	4.77
**Percentage**	0.83	0.06	2.33	0.33	2.38	94.08

* *m*/*z* for [M − H]^−^.

**Table 3:** Batch summary for isolation of OA and DTX1 from *P. lima*, as assessed by LC-HRMS (Section 3.5.1).

	Weight (mg)	Clean-up (100 × (W_i_ − W_f_)/W_i_) (%)	OA (mg)	Step Recovery (%)	Purity (%)	DTX1 (mg)	Step Recovery (%)	Purity (%)
Culture (4.5 L)			3.3			10.5		
Step 1 (HP-20 resin extract)	322		3.2		1.0	10.2		3.2
Step 2 (alumina/SPE)	47.2	85.3	2.9	90.6	6.1	8.9	87.3	18.9
Step 3 (LH-20)	19.5	58.7	2.8	96.6	14.4	8.5	95.5	43.6
Step 4 (semi-prep HPLC)			2.5	89.3	* 98.5	8.0	94.1	^ 97.3
% Recovery			75.8			76.2		

W_i_ = initial weight, W_f_ = final weight, * See Figure S11, ^ See Figure S14.

**Table 4:** LC-HRMS (Section 3.5.2) analysis of purified OA (after NMR analysis, Figure S12).

Toxin	OA + MeOH		DTX1 Isomer	DTX1	OA Isomer	19-*epi*-OA	OA
Accurate mass (*m/z*) *	835.4822		817.4744	817.4744	803.4577	803.4587	803.4588
**Δ *m/z* (ppm)**	−3.3		0.0	0.0	−1.3	0.0	0.1
**Retention time (min)**	3.95	4.36	4.65	5.32	4.34	4.43	4.54
**Percentage**	0.08	0.04	0.07	0.18	0.33	1.38	97.91

* *m*/*z* for [M − H]^−^.

**Table 5:** LC-HRMS (Section 3.5.2) analysis of purified DTX1 (after NMR analysis, Figure S15).

Toxin	DTX1 + MeOH	7-deoxyOA	DTX1 Isomer	19-*epi*-DTX1	DTX1
**Accurate mass (*m/z*)** *	849.5032	787.4612	817.4731	817.4757	817.4746
**Δ *m/z* (ppm)**	3.0	−3.3	−1.5	1.6	0.3
**Retention time (min)**	4.46	5.39	4.60	5.07	5.31
**Percentage**	0.08	0.09	0.60	2.04	97.19

* *m*/*z* for [M − H]^−^.

## Supplementary Materials

The following are available online at https://www.mdpi.com/1660-3397/18/12/647/s1, Figure S1: LC-UV clean-up of DTX2 using alumina and sax chromatography, Figures S2 and S3: Purity analysis of DTX2 by LC-HRMS (acidic and neutral methods), Figure S4: Purity analysis of DTX2 by LC-UV, Figure S5: Kinetic analysis of 19-*epi*-DTX2 in acidic MeOH, Figures S6–S10: 1H NMR spectra of OA, DTX1, and DTX2 in CD_3_OD, Figures S11 and S12: Purity analysis of OA by LC-HRMS (acidic and neutral methods), Figure S13: Purity analysis of OA by LC-UV, Figures S14 and S15: Purity analysis of DTX1 by LC-HRMS (acidic and neutral methods), Figure S16: Purity analysis of DTX1 by LC-UV.
