# Supplementary material for: Improved Isolation Procedures for Okadaic Acid Group Toxins from Shellfish (Mytilus edulis) and Microalgae (Prorocentrum lima)
Source: Mar Drugs. 2020 Dec 16;18(12):647. doi: 10.3390/md18120647 (PMC7766028; doi:10.3390/md18120647)
Supplement: Supplementary file 1 [file marinedrugs-18-00647-s001.pdf]

Improved Isolation Procedures for Okadaic Acid Group Toxins from Shellfish (*Mytilus edulis*) and Microalgae (*Prorocentrum lima*)

Jane Kilcoyne, Stephen Burrell, Cíara Nulty, Rafael Salas, Elliott J. Wright, Isabelle Rajotte, Christopher O. Miles

Contents

Figure S1. LC-UV showing difference in clean up of DTX2 between alumina and sax chromatography S2
Figure S2. Purity analysis of DTX2 by LC-HRMS (acidic method) S3
Figure S3. Purity analysis of DTX2 by LC-HRMS (neutral method) S4
Figure S4. Purity analysis of DTX2 by LC-UV S5
Figure S5. Kinetic analysis and LC-HRMS/MS chromatograms from isomerization of 19-epi-DTX2 in acidic MeOH S6
Figure S6. 1H NMR spectra (0.65–6.00 ppm) of OA, DTX1, and DTX2 in CD3OD S7
Figure S7. 1H NMR spectra (0.65–2.40 ppm) of OA, DTX1, and DTX2 in CD3OD S8
Figure S8. 1H NMR spectra (3.10–4.70 ppm) of OA, DTX1, and DTX2 in CD3OD S9
Figure S9. 1H NMR spectra (5.00–6.00 ppm) of OA, DTX1, and DTX2 in CD3OD S10
Figure S10. 1H NMR spectra (5.40–5.95 ppm) of OA, DTX1, and DTX2 in CD3OD S11
Figure S11. Purity analysis of OA by LC-HRMS (acidic method) S12
Figure S12. Purity analysis of OA by LC-HRMS (neutral method) S13
Figure S13. Purity analysis of OA by LC-UV S14
Figure S14. Purity analysis of DTX1 by LC-HRMS (acidic method) S15
Figure S15. Purity analysis of DTX1 by LC-HRMS (neutral method) S16
Figure S16. Purity analysis of DTX1 by LC-UV S17

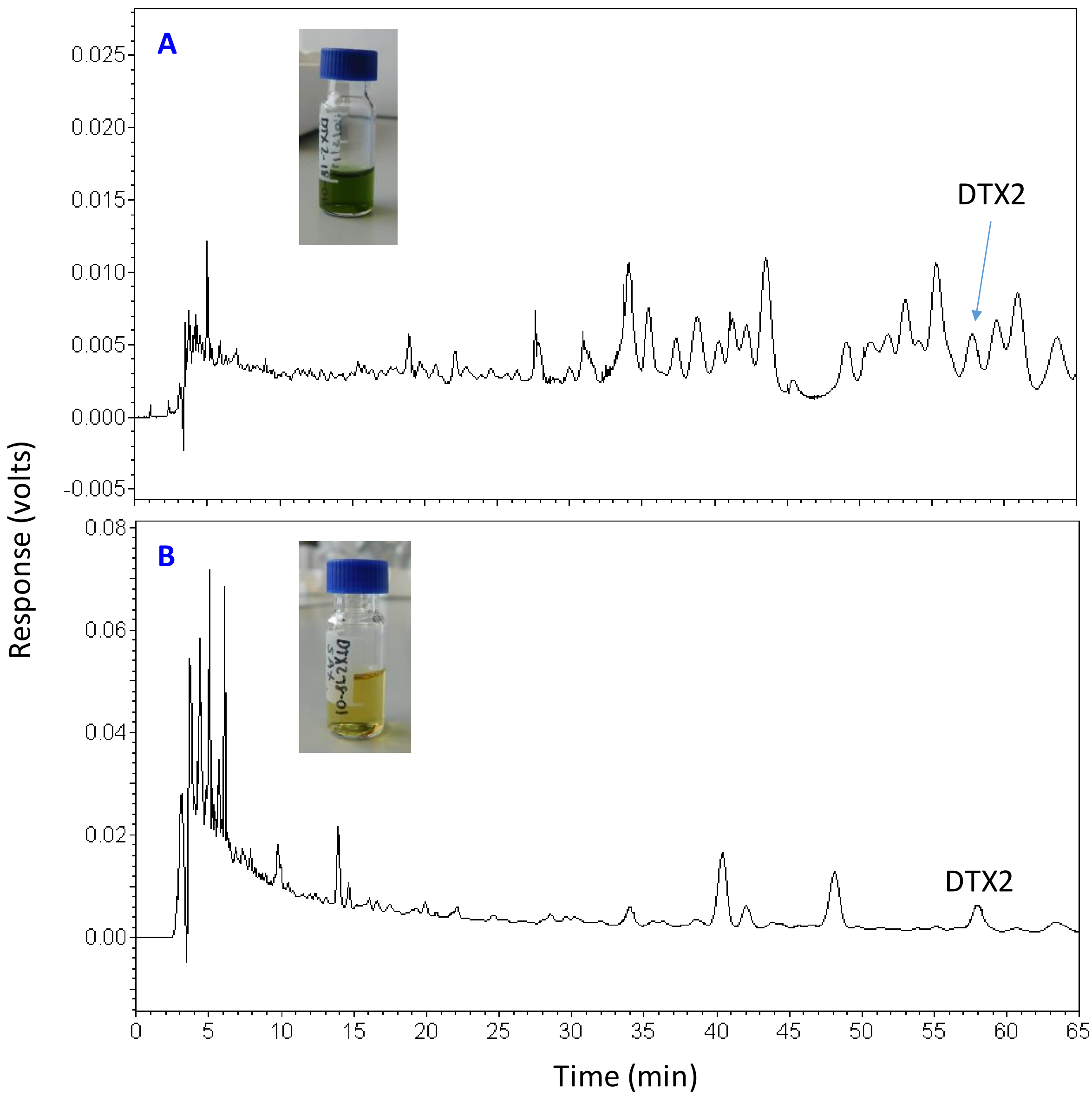

Figure S1. LC-UV (210 nm) chromatography showing difference in clean up between A) alumina (1 g loaded onto 30 g alumina) and B) SAX chromatography (3.4 g loaded onto 11.5 g SAX).

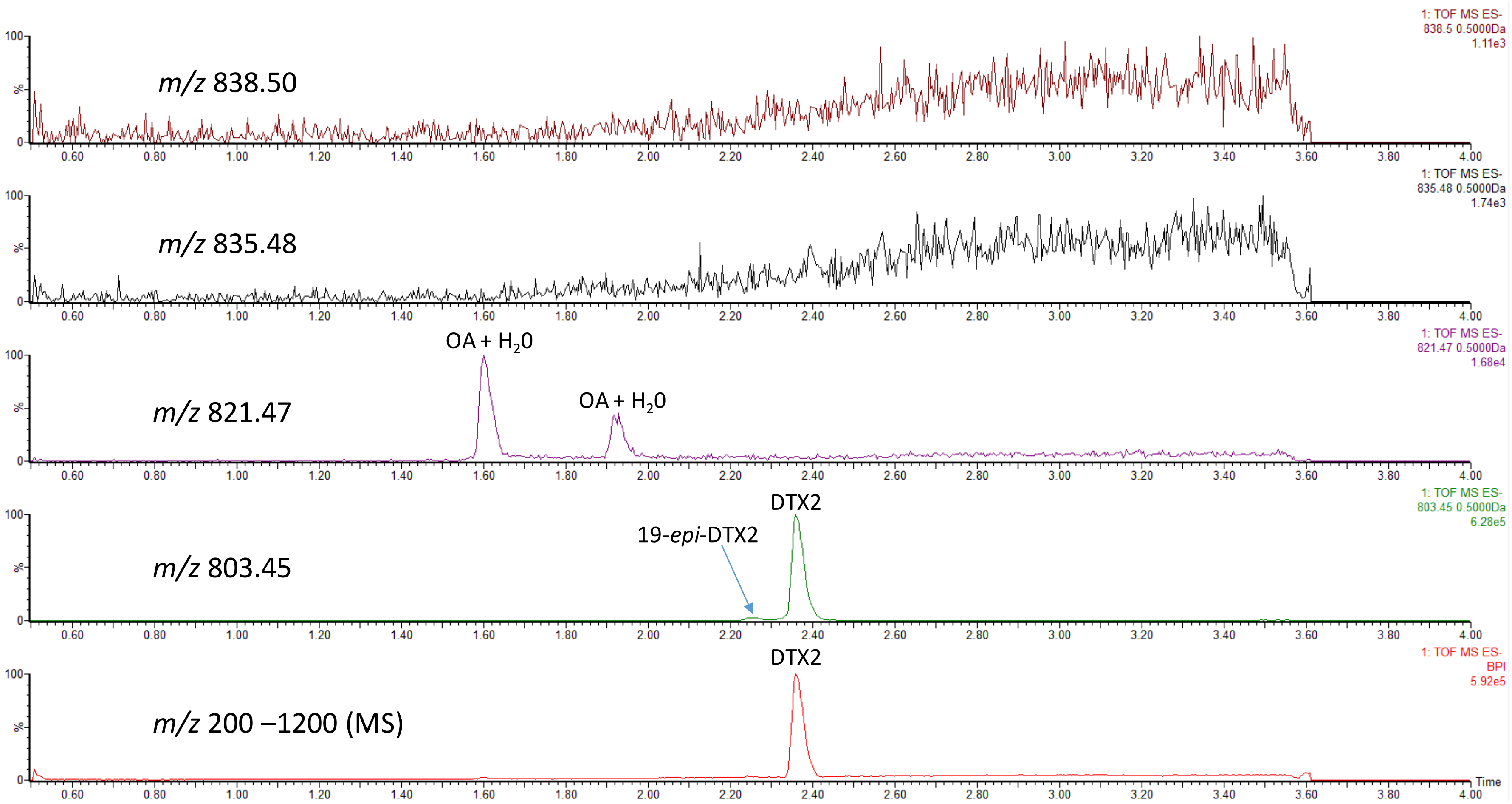

Figure S2. Purity analysis of DTX2 by LC-HRMS (acidic mobile phase, section 3.5.1).

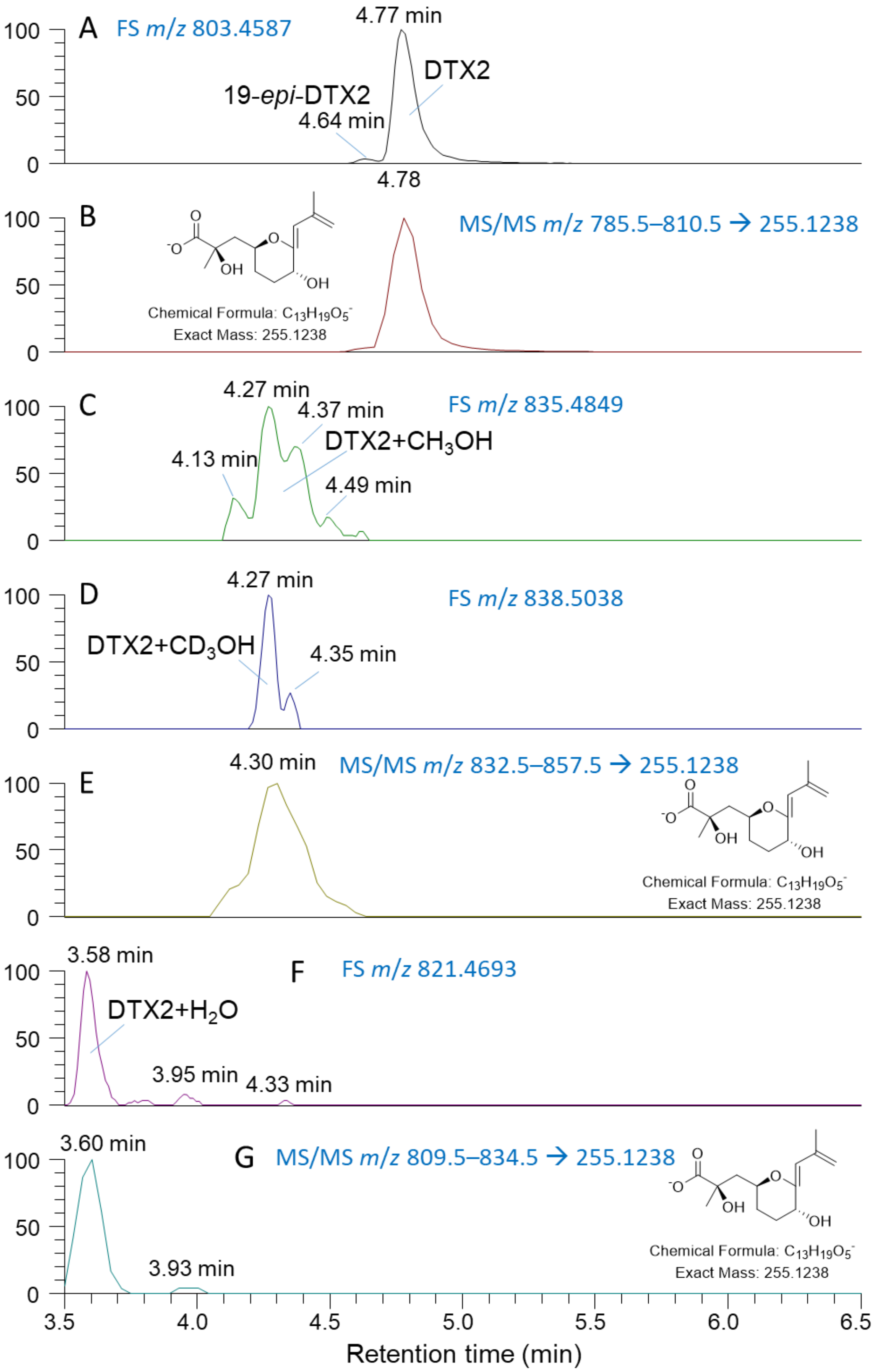

Figure S3. LC-HRMS/MS (neutral mobile phase, section 3.5.2) chromatograms of the purified DTX2 after NMR analysis, showing full scan (FS) chromatograms A, C, D and F extracted at the exact *m*/*z* values for DTX2, DTX2 + MeOH, DTX2 + CD_3OH, and DTX2 + H_2O (± 5 ppm), respectively, above the chromatograms of the corresponding DIA windows B, E and G extracted for product ions at *m*/*z* 255.1238 (± 5 ppm).

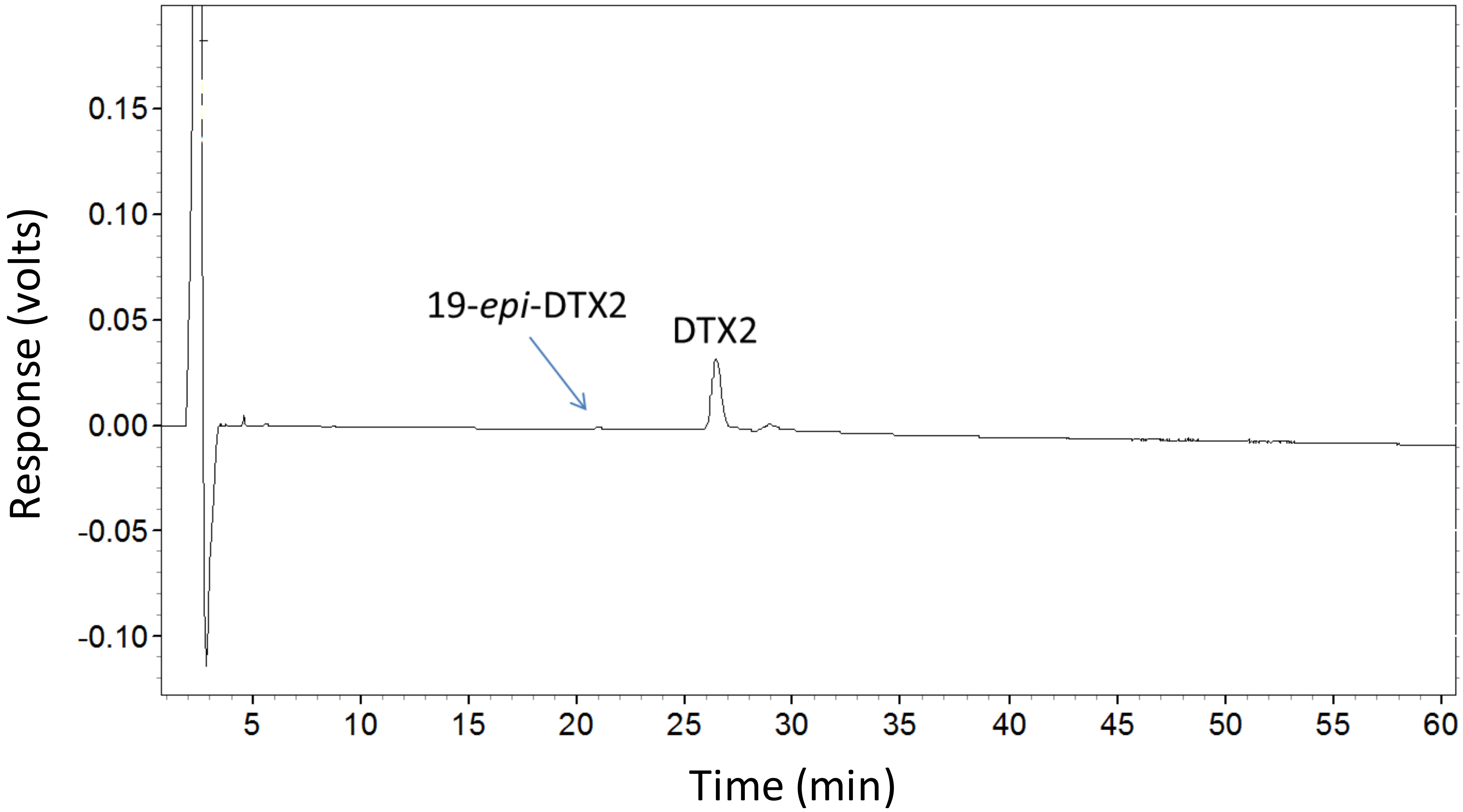

Figure S4. Purity analysis of DTX2 by LC-UV (210 nm, section 3.6).

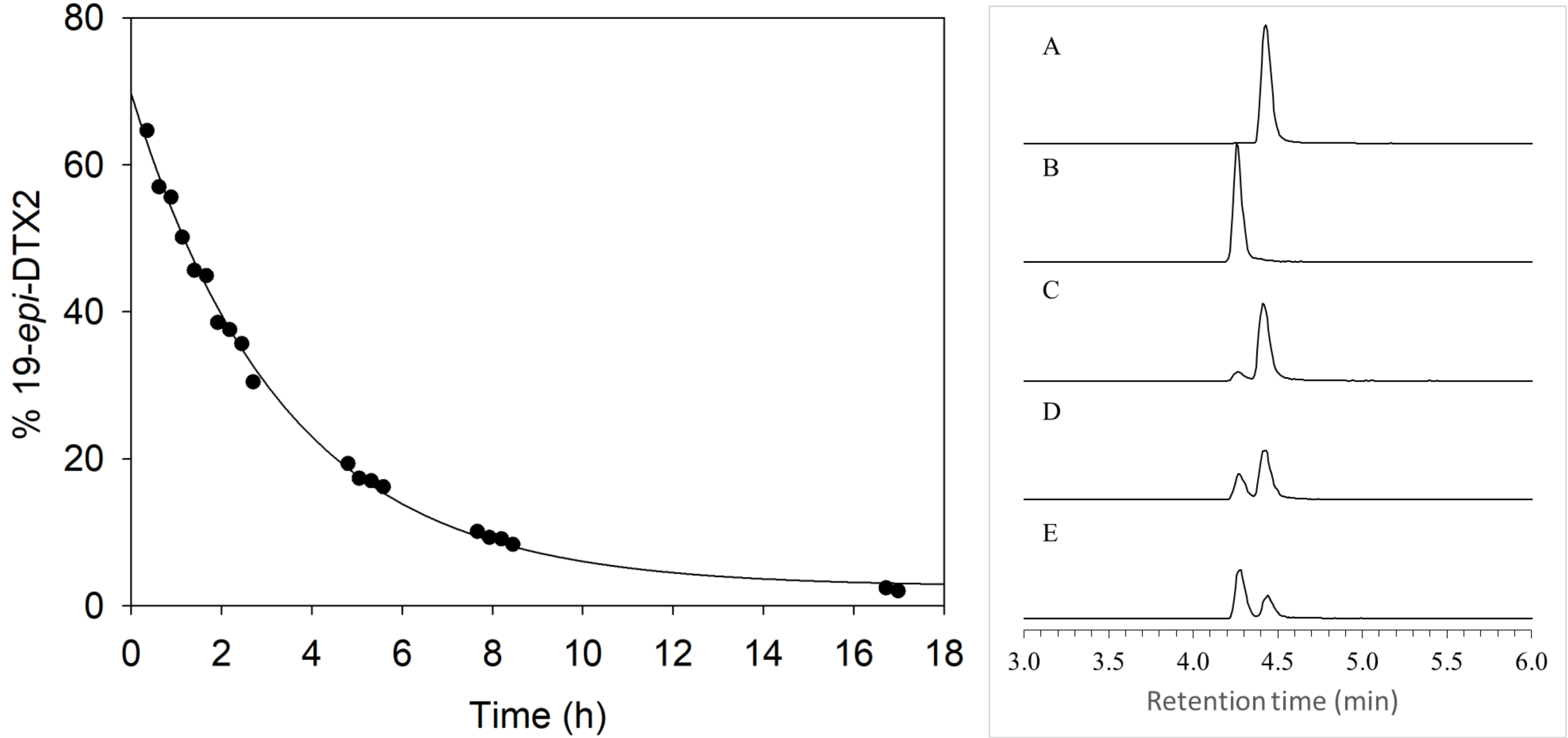

Figure S5. Left, percentage of 19-*epi*-DTX2 (as a percentage of the sum of 19-*epi*-DTX2 and DTX2) remaining versus time during incubation at 10 ℃ in MeOH containing ~1% formic acid. The fitted line is a 3-parameter exponential decay curve with $t_{½}$ 2.3 h (SE ±0.1) and final equilibrium concentration 2.6% (SE ±0.7). Right, LC–MS/MS chromatograms (*m*/*z* 803.5→255.1) of: A) NRC-CRM-DTX2b; B) purified 19-*epi*-DTX2, and; C–E) 19-*epi*-DTX2 incubated for 7.68, 2.70, and 0.35 h in MeOH containing ~1% formic acid, which formed part of the kinetic analysis shown in the graph on the left.

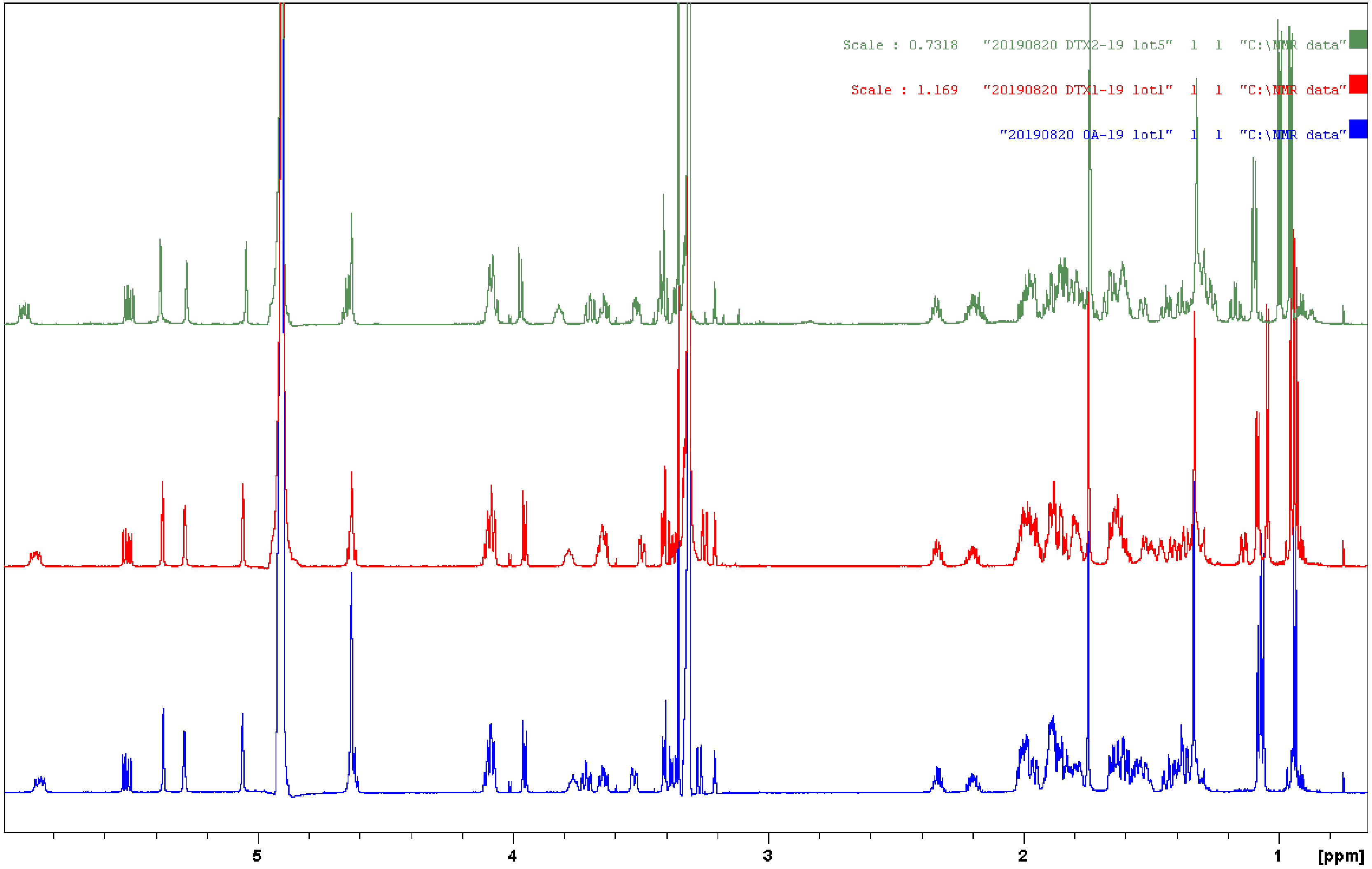

Figure S6. ^{1}H NMR spectra (0.65–6.00 ppm) of: top, DTX2 (green); middle, DTX1 (red), and; OA (blue) in CD_3OD. Spectral intensities are scaled so that spectra have the same peak heights for the olefinic resonances at 5.0–5.6 ppm.

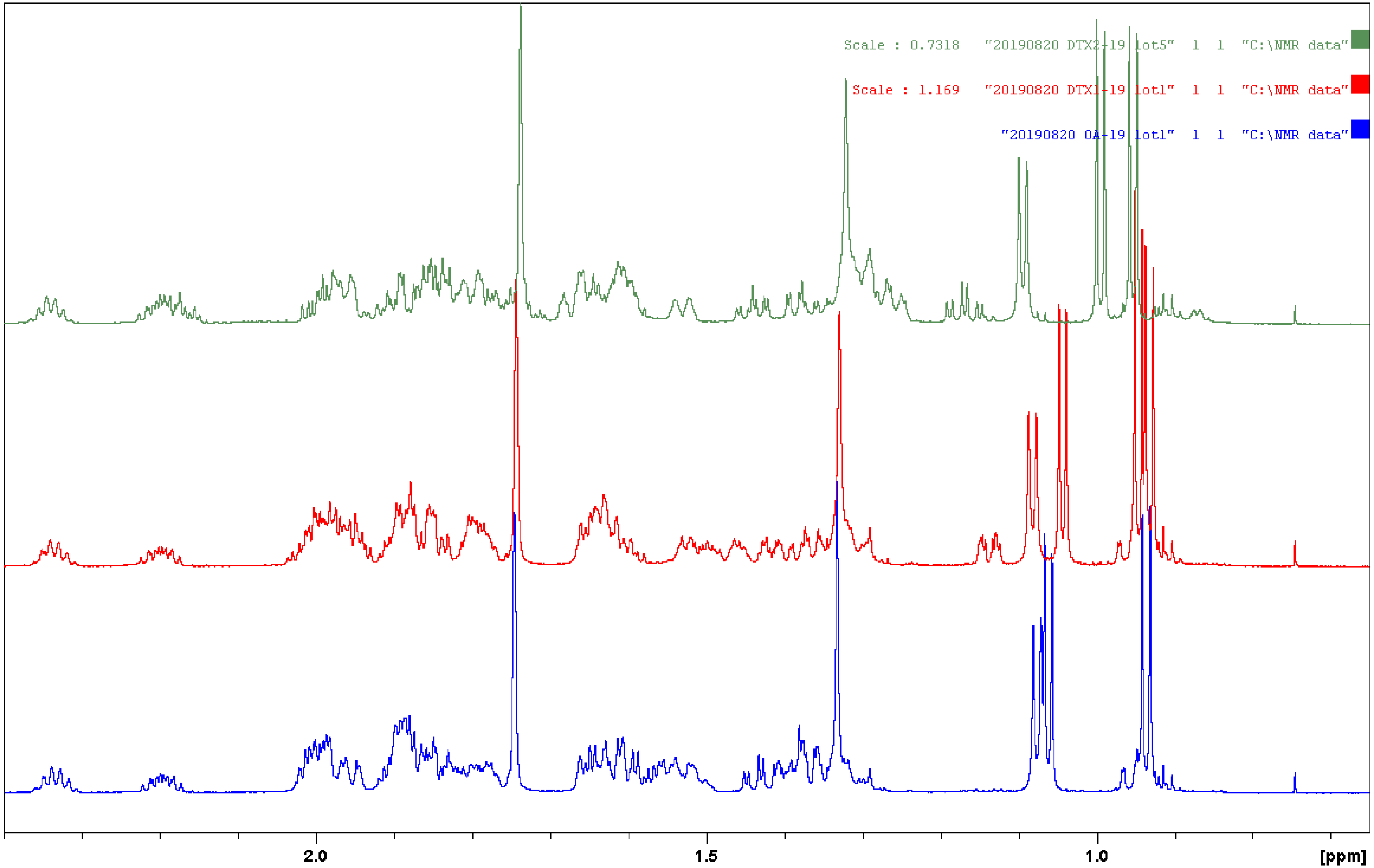

Figure S7. Expansion (0.65–2.40 ppm) of the ^{1}H NMR spectra shown in Figure S6. Top, DTX2 (green); middle, DTX1 (red), and; OA (blue) in CD_3OD.

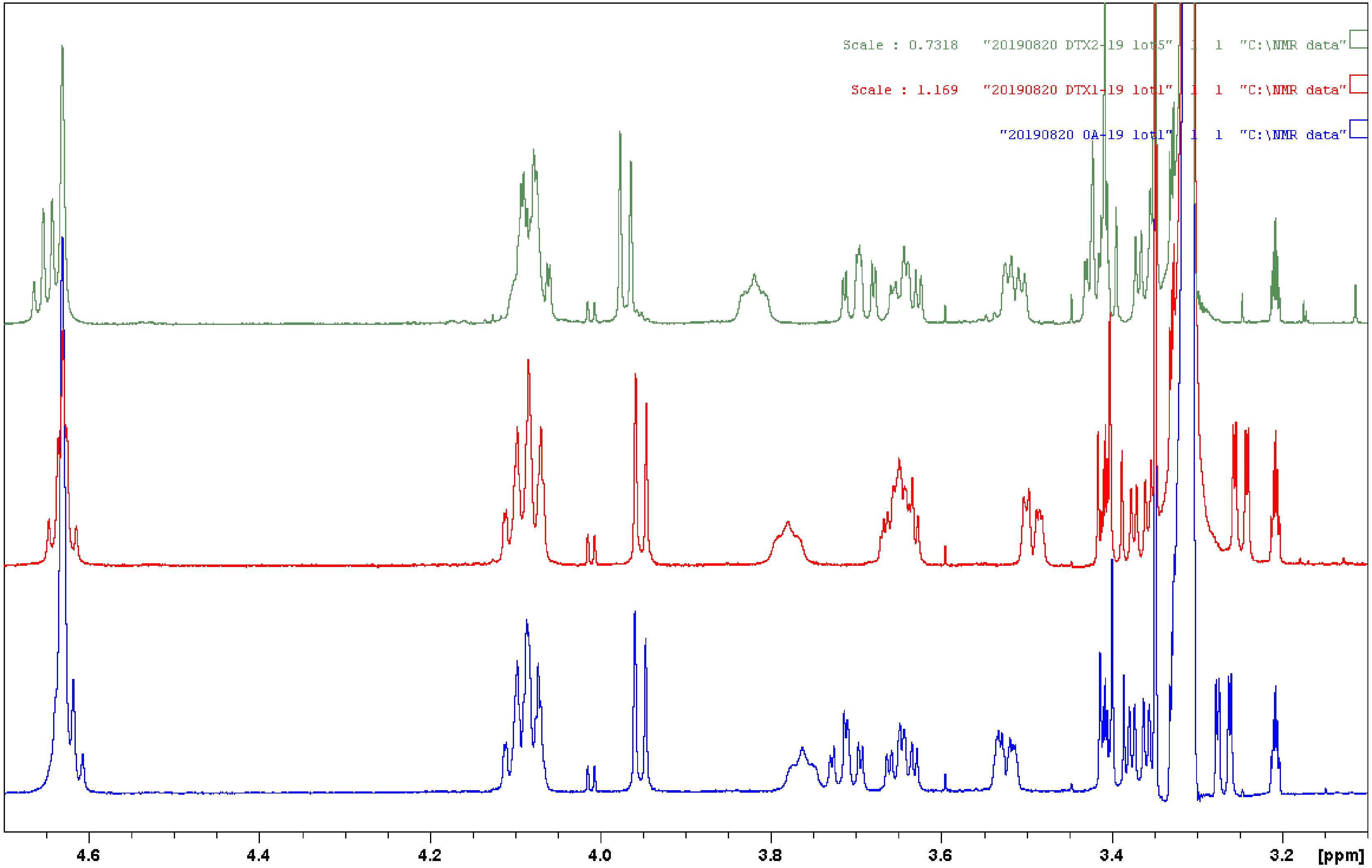

Figure S8. Expansion (3.10–4.70 ppm) of the ^{1}H NMR spectra shown in Figure S6. Top, DTX2 (green); middle, DTX1 (red), and; OA (blue) in CD_3OD.

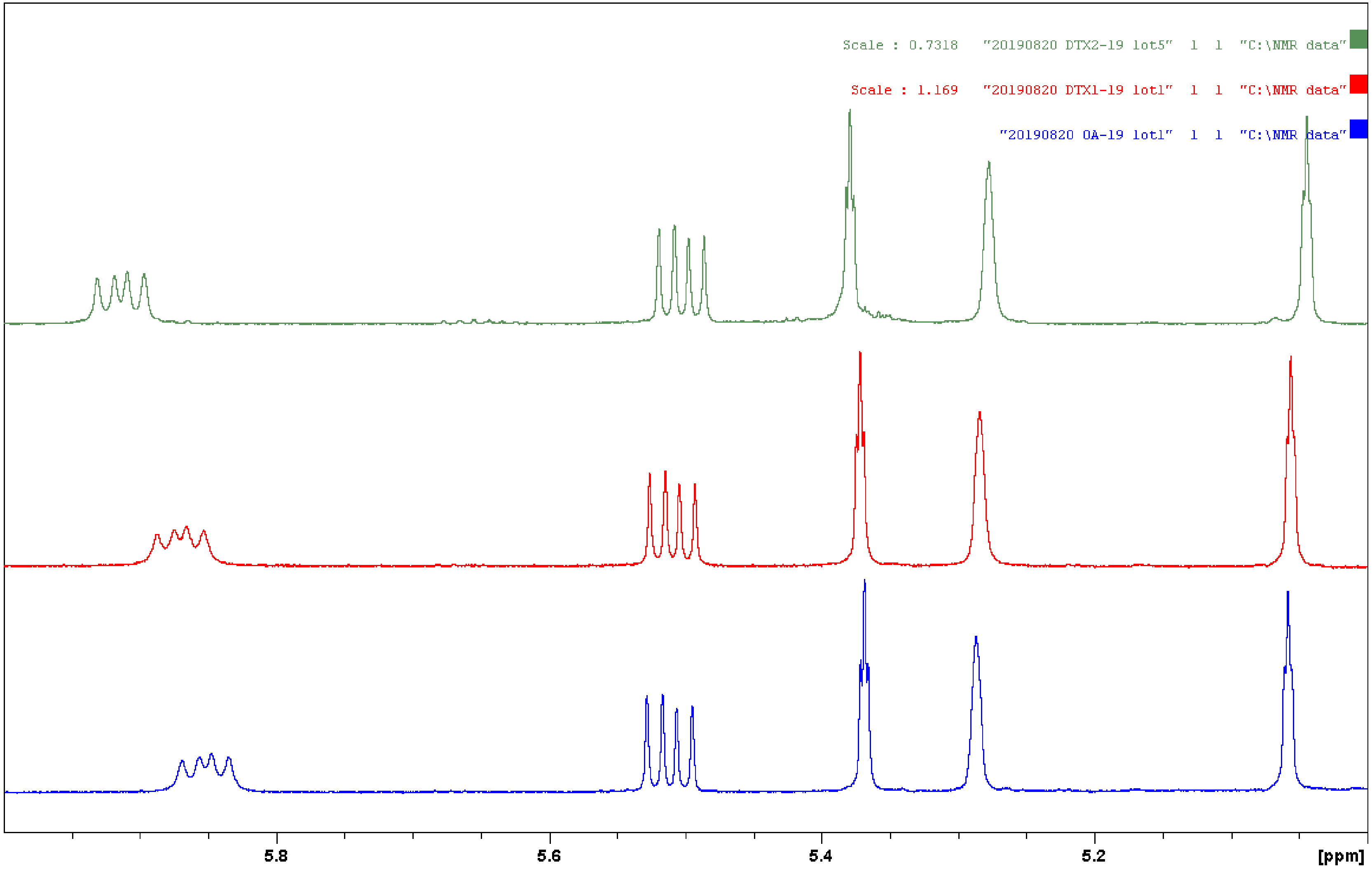

Figure S9. Expansion (5.00–6.00 ppm) of the ^{1}H NMR spectra shown in Figure S6. Top, DTX2 (green); middle, DTX1 (red), and; OA (blue) in CD_3OD.

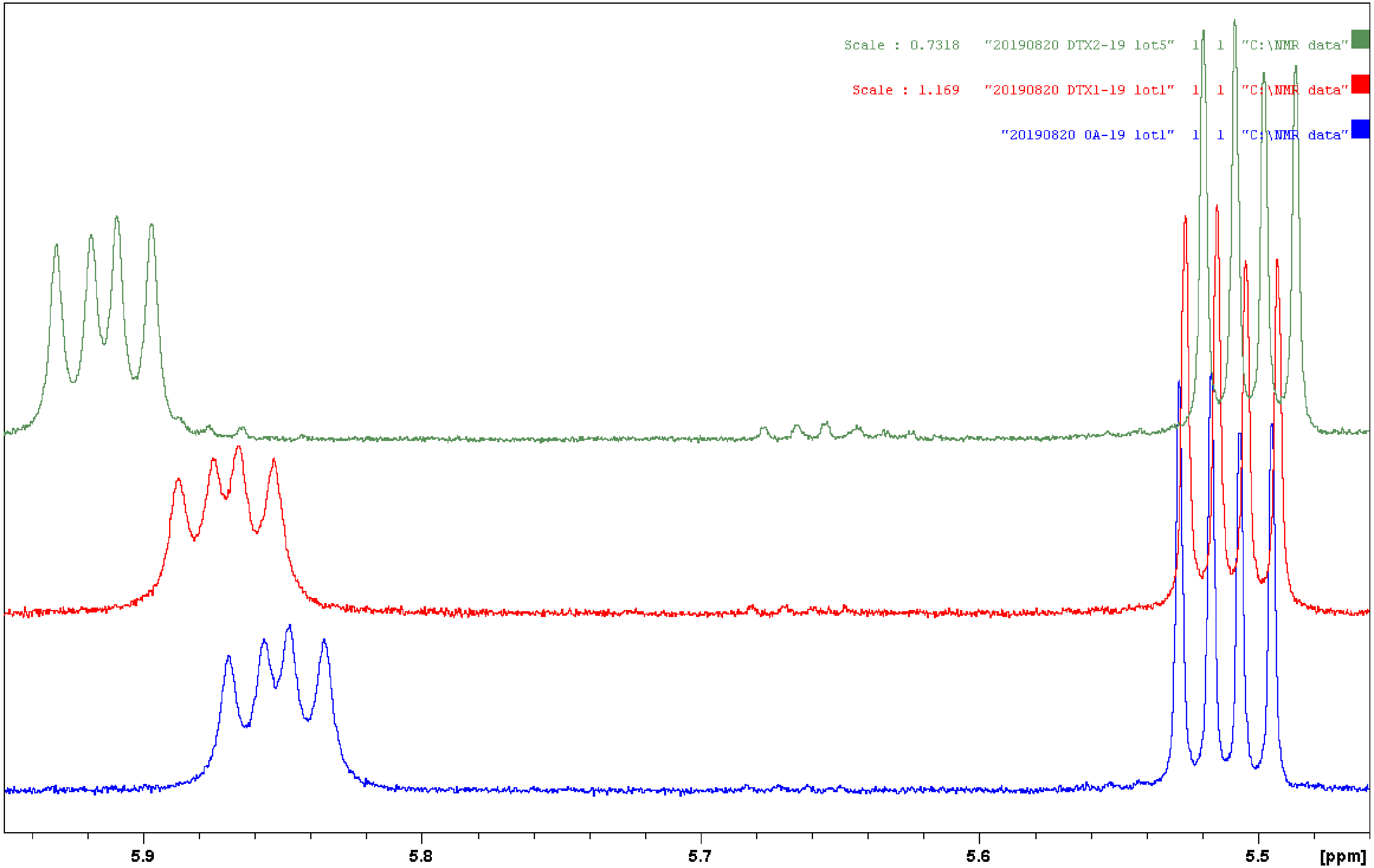

Figure S10. Expansion (5.40–5.95 ppm) of the ^{1}H NMR spectra shown in Figure S6. Top, DTX2 (green); middle, DTX1 (red), and; OA (blue) in CD_3OD. Signs of signals from possible isomers and analogues are visible at ~5.66 ppm, particularly in DTX2 (~5%). Other minor resonances at 5.88 ppm (DTX2) and at 5.55 may also be due to structurally-related impurities.

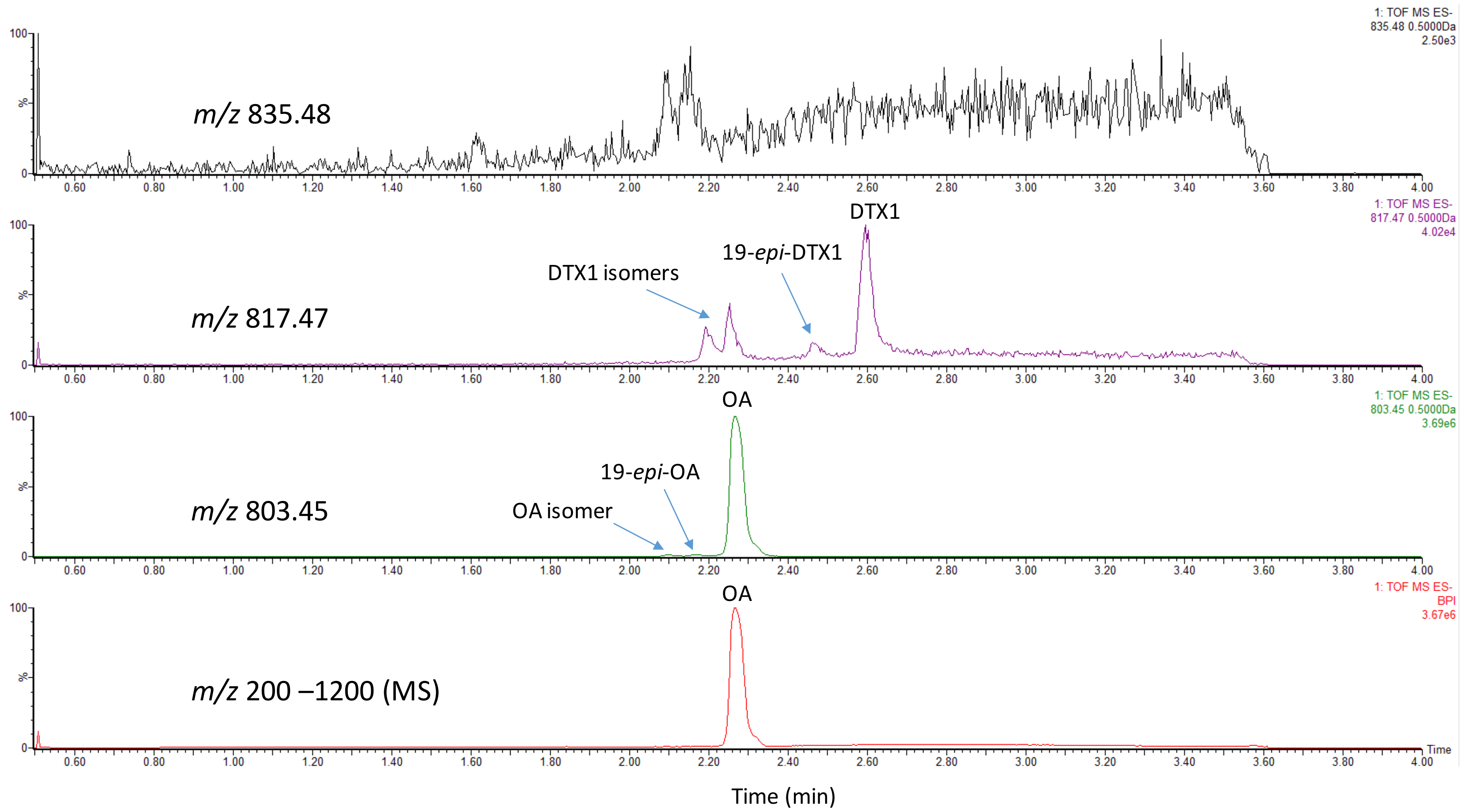

Figure S11. Purity analysis of OA by LC-HRMS (acidic mobile phase, section 3.5.1).

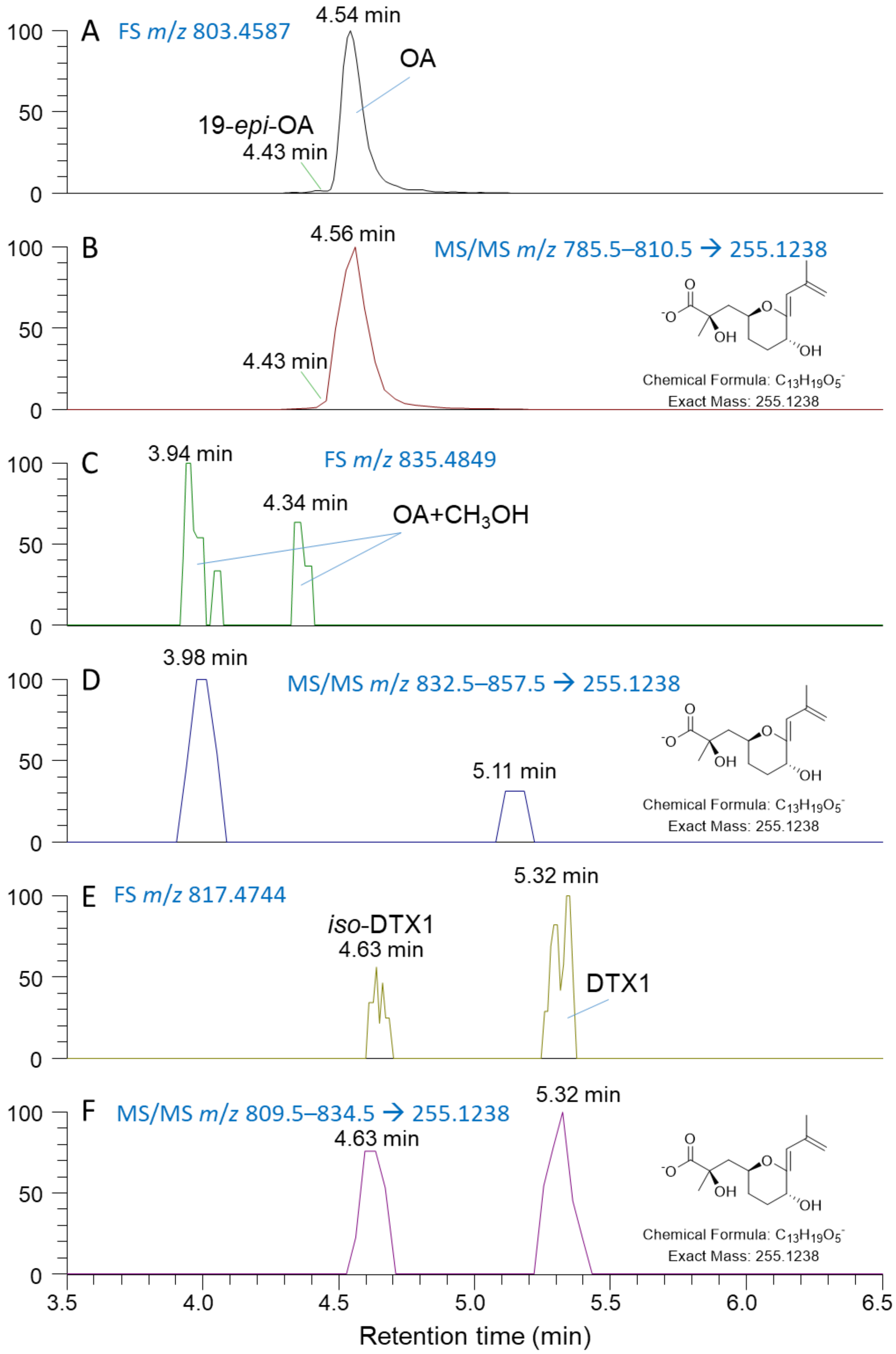

Figure S12. LC-HRMS/MS (using neutral mobile phase, section 3.5.2) chromatograms of the purified OA after NMR analysis, showing full scan (FS) chromatograms A, C, and E extracted at the exact *m*/*z* values for OA, OA + MeOH, and DTX1/*iso*-DTX1 (± 5 ppm), respectively, above the chromatograms of the corresponding DIA windows B, D and F extracted for product ions at *m*/*z* 255.1238 (± 5 ppm).

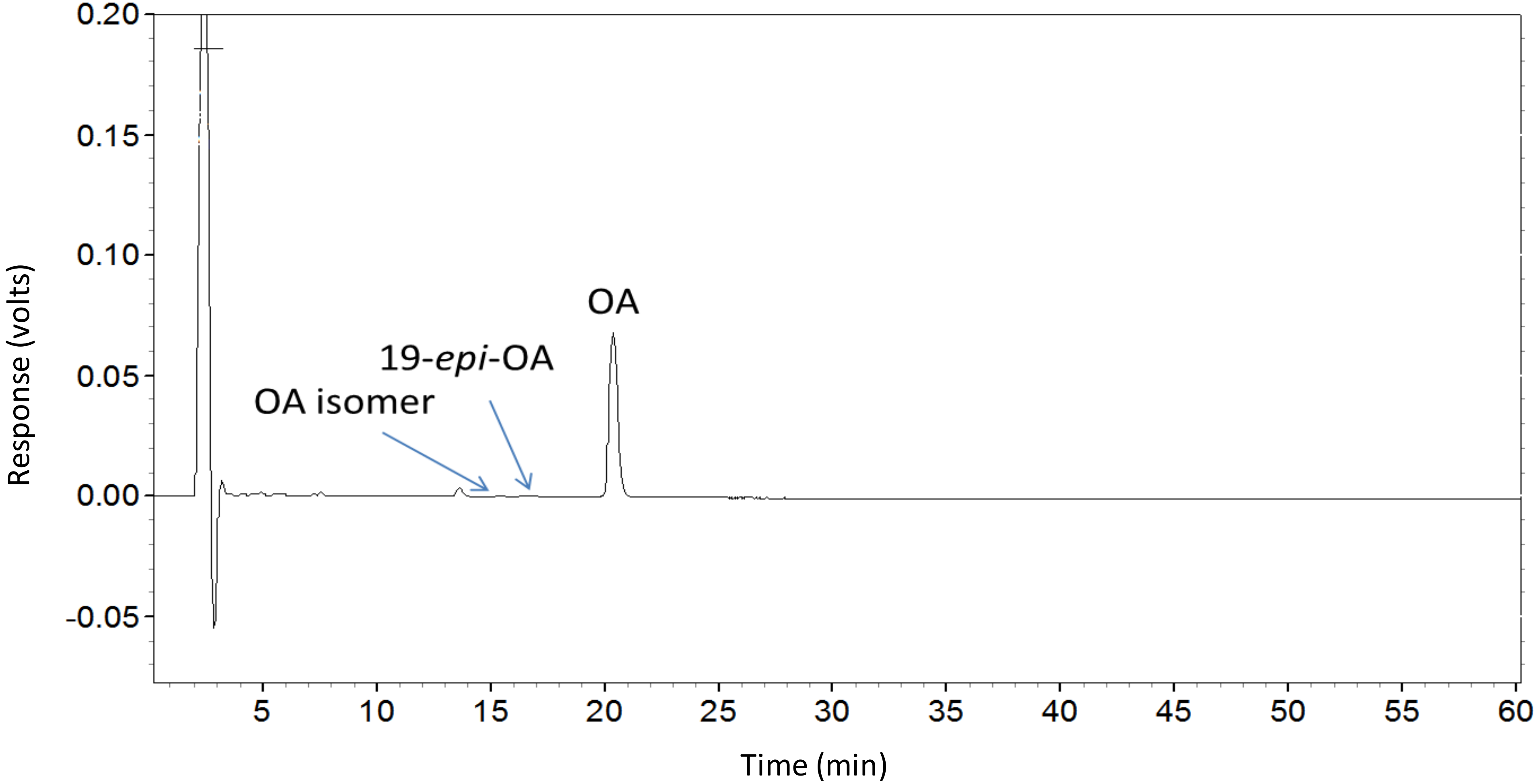

Figure S13. Purity analysis of OA by LC-UV (210 nm, section 3.6).

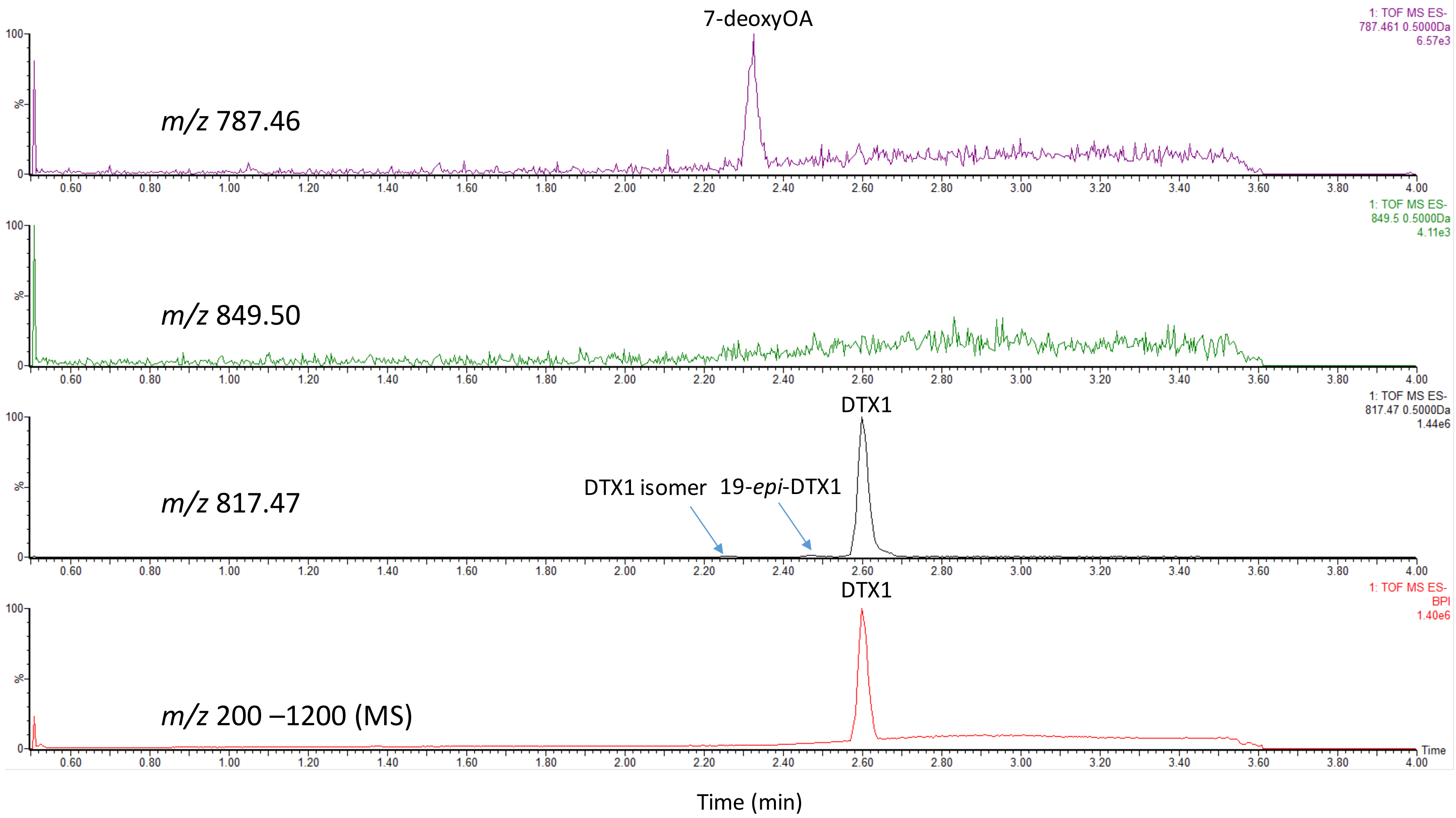

Figure S14. Purity analysis of DTX1 by LC-HRMS (acidic mobile phase, section 3.5.1).

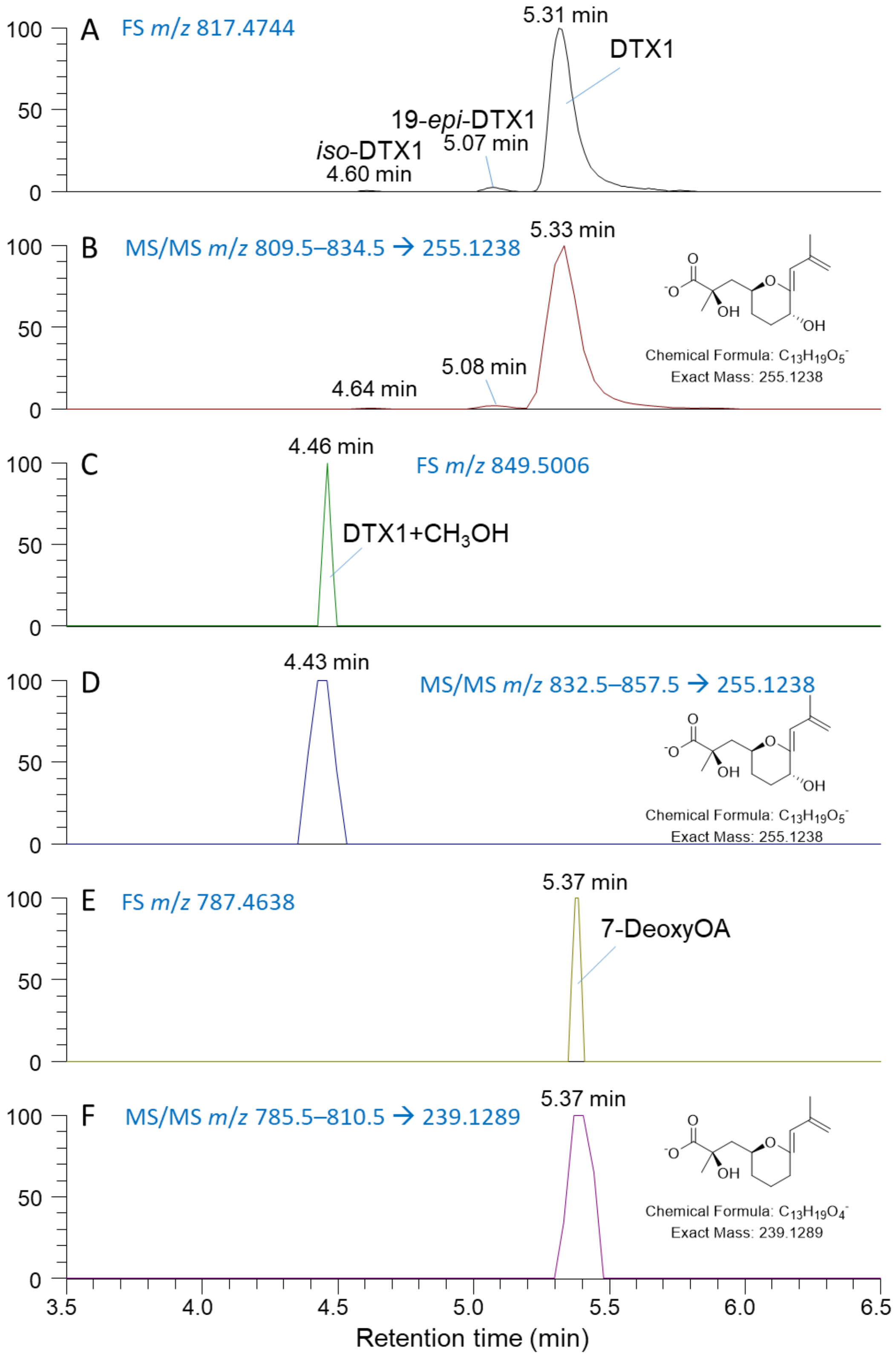

Figure S15. LC-HRMS/MS (neutral mobile phase, section 3.5.2) chromatograms of the purified DTX1 after NMR analysis, showing full scan (FS) chromatograms A, C, and E extracted at the exact *m*/*z* values for DTX1, DTX1 + MeOH, and 7-deoxyOA (±5 ppm), respectively, above the chromatograms of the corresponding DIA windows B, and D extracted for product ions at *m*/*z* 255.1238 and F extracted for *m*/*z* 239.1289 (±5 ppm).

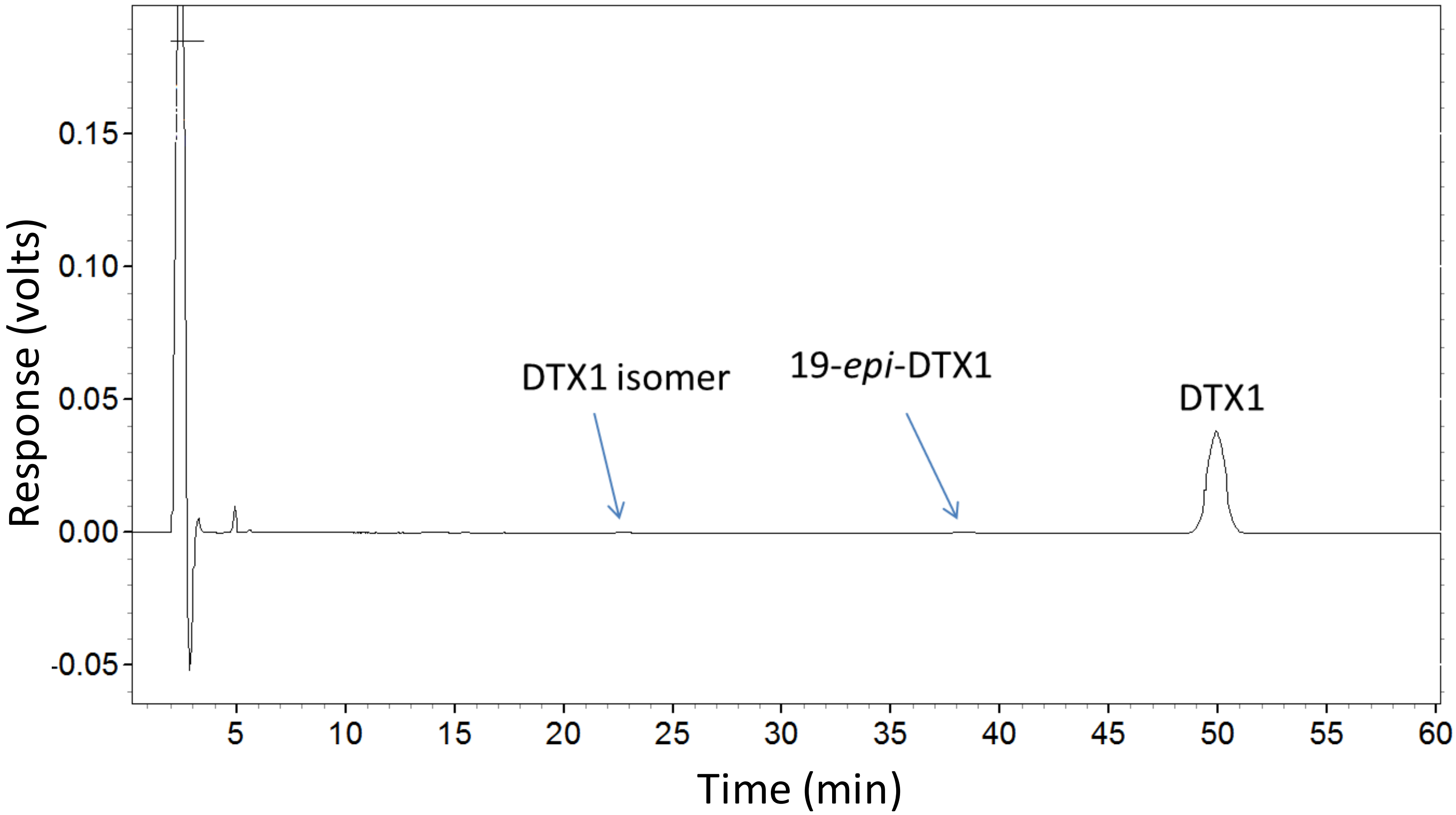

Figure S16. Purity analysis of DTX1 by LC-UV (210 nm, section 3.6).
